# Screen media activity does not displace other recreational activities among 9–10 year-old youth: a cross-sectional ABCD study®

**DOI:** 10.1186/s12889-020-09894-w

**Published:** 2020-11-25

**Authors:** Briana Lees, Lindsay M. Squeglia, Florence J. Breslin, Wesley K. Thompson, Susan F. Tapert, Martin P. Paulus

**Affiliations:** 1 The Matilda Centre for Research in Mental Health and Substance Use, University of Sydney, Level 6 Jane Foss Russell Building, G02, Camperdown, NSW 2006 Australia; 2 Department of Psychiatry and Behavioral Sciences, Medical University of South Carolina, Addiction Sciences Division, 171 Ashley Ave, Charleston, SC 29425 USA; 3 Laureate Institute for Brain Research, 6655 S Yale Ave, Tulsa, OK 74136 USA; 4 Division of Biostatistics, Department of Family Medicine and Public Health, University of California San Diego, 9500 Gilman Dr, La Jolla, CA 92093 USA; 5 Department of Psychiatry, University of California San Diego, 9500 Gilman Dr, La Jolla, CA 92093 USA

**Keywords:** Screen media, Social media, Sport, Physical activity, Recreational activities, Hobbies, Displacement hypothesis, Children

## Abstract

**Background:**

Screen media is among the most common recreational activities engaged in by children. The displacement hypothesis predicts that increased time spent on screen media activity (SMA) may be at the expense of engagement with other recreational activities, such as sport, music, and art. This study examined associations between non-educational SMA and recreational activity endorsement in 9–10-year-olds, when accounting for other individual (i.e., cognition, psychopathology), interpersonal (i.e., social environment), and sociodemographic characteristics.

**Methods:**

Participants were 9254 youth from the Adolescent Brain Cognitive Development Study®. Latent factors reflecting SMA, cognition, psychopathology, and social environment were entered as independent variables into logistic mixed models. Sociodemographic covariates included age, sex, race/ethnicity, education, marital status, and household income. Outcome variables included any recreational activity endorsement (of 19 assessed), and specific sport (swimming, soccer, baseball) and hobby (music, art) endorsements.

**Results:**

In unadjusted groupwise comparisons, youth who spent more time engaging with SMA were less likely to engage with other recreational activities (*p*s < .001). However, when variance in cognition, psychopathology, social environment, and sociodemographic covariates were accounted for, most forms of SMA were no longer significantly associated with recreational activity engagement (*p* > .05). Some marginal effects were observed: for every one SD increase in time spent on games and movies over more social forms of media, youth were at lower odds of engaging in recreational activities (adjusted odds ratio = 0·83, 95% CI 0·76–0·89). Likewise, greater general SMA was associated with lower odds of endorsing group-based sports, including soccer (0·93, 0·88–0·98) and baseball (0·92, 0·86–0·98). Model fit comparisons indicated that sociodemographic characteristics, particularly socio-economic status, explained more variance in rates of recreational activity engagement than SMA and other latent factors. Notably, youth from higher socio-economic families were up to 5·63 (3·83–8·29) times more likely to engage in recreational activities than youth from lower socio-economic backgrounds.

**Conclusions:**

Results did not suggest that SMA largely displaces engagement in other recreational activities among 9–10-year-olds. Instead, socio-economic factors greatly contribute to rates of engagement. These findings are important considering recent shifts in time spent on SMA in childhood.

**Supplementary Information:**

The online version contains supplementary material available at 10.1186/s12889-020-09894-w.

## Introduction

Childhood is a critical period for the development and establishment of behaviors and attitudes that continue into adult life [1]. Children and adolescents who have partaken in a variety of physical and recreational activities are much more active as adults [2], and a lifestyle that includes regular physical and social activity has been associated with numerous immediate and long-term health benefits. These include lower risk of mental health issues, obesity, and cardiovascular disease risk factors [3]. Conversely, sedentary behavior is predictive of poor metabolic and physical health, and social wellbeing in childhood [4]. Children and adolescents report a multitude of sedentary behaviors, some of which are necessary and/or should not be discouraged (e.g., homework, hobbies). However, much of their sedentary time involves non-educational screen media activity (e.g., television watching, computer gaming, social media engagement). The amount of leisure time spent by children and adolescents online has doubled in the past decade [5]. Children spend up to 50% of their time after school on screens, including cell phones, tablets, computers, gaming consoles, and televisions [6]. Over 94% of children aged 11 years use a cell phone [7] and approximately 85% engage in electronic gaming [8]. Therefore, it is important that research examine the associated outcomes of this shift in leisure time spent on screen media activity (SMA) in childhood.

The displacement hypothesis predicts that SMA and other activities compete for leisure time, where screen time might be at the expense of other recreational activity involvement such as sport and other hobbies, which are potentially more beneficial for health and cognitive development [9]. For the most part, previous studies investigating this hypothesis have focused on the impacts of SMA on physical activity and have reported inconsistent findings. Some studies have reported moderate inverse relationships between SMA and physical activity in adolescents, where greater SMA use has been associated with lower activity [10–13]. Conversely, two systematic reviews including samples of up to 31,022 youth have found a common “technoactive” cluster of young people who engage in high levels of sports and SMA [14, 15]. However, a cross-national study from 39 countries with a very large sample size (*n* = 200,615) reported no consistent association between SMA time and physical activity in youth aged 11, 13, and 15 years [16]. Likewise, a recent systematic review of reviews [4] and a meta-analysis of 163 studies [17] have found very little empirical evidence to suggest that playing digital games, using a computer, and watching television competes with physical activity involvement in children and adolescents. Overall, results on the interdependence of SMA and recreational physical activity involvement in childhood are inconsistent.

Exploring different types of recreational activities (e.g., sports, music, art) and different forms of media (e.g., television viewing, electronic gaming, cell phones, tablets, computers, social media-related SMA), using data-driven techniques which group and characterize similar patterns of behavior, may be useful [4]. Associations may differ for various forms of SMA and recreational activities. Additionally, other individual, interpersonal, and sociodemographic factors are likely to play a role in these relations. For instance, when compared to high levels of social media messaging, greater television viewing or gaming may be associated with social isolation, depression, anxiety, and self-injurious behavior in children and adolescents [11, 18, 19]. In turn, this may decrease interest and involvement in group-based sports and clubs, or vice versa [11, 19]. Yet, analysis of these different activity settings and types of SMA use, as well as sociodemographic, cognitive, social, and psychopathology factors likely impacting these associations, are uncommon. Moreover, many children also spend their leisure time engaging with hobbies other than physical activity, such as music and art. In contrast to research on associations between SMA and physical activity, studies on other hobbies are particularly sparse.

In light of this, the current research aimed to examine unique associations between various data-driven forms of non-educational SMA use and recreational activities including sports, music, and art, when accounting for other individual (i.e., cognition, psychopathology), interpersonal (i.e., social environment), and sociodemographic factors. Cross-sectional data were utilized from a large participant sample of children aged 9 to 10 years, collected in 2016 and 2017.

## Methods

### Participants

This study used baseline cross-sectional data from participants aged 9 to 10 years included in the Adolescent Brain Cognitive Development (ABCD) Data Release 2.0.1. The Adolescent Brain Cognitive Development Study is the largest long-term study of child health in the United States, with 21 research sites across the nation. A probability sample was recruited through school systems with school selection informed by sex, race and ethnicity, socio-economic status, and urbanicity [20]. Written informed consent and assent were obtained from a parent or legal guardian and the child, respectively. All procedures were approved by an Institutional Review Board. Of 11,875 participants enrolled, 9254 had complete data on all relevant measures and were eligible to be included in the current study.

### Outcome measure

**Fig. 1:**
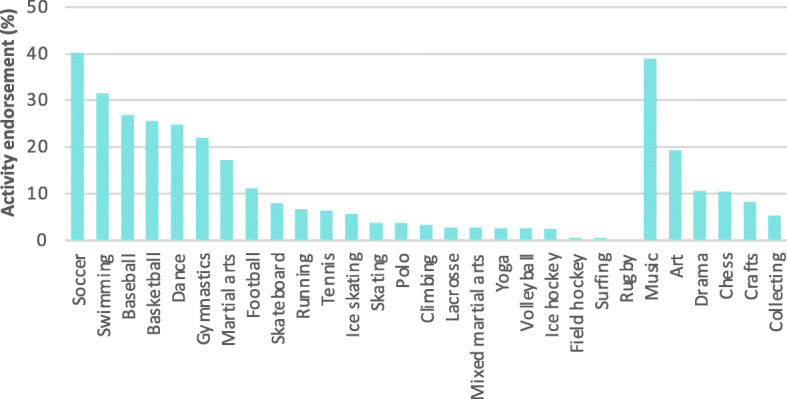
Parent-reported endorsement rates of 29 recreational activities

Youth participation in a variety of organized recreational activities was assessed via The Sports and Activities Involvement Questionnaire [21]. Parents reported on the frequency, duration, and type of activity their child participates in, including physical activity, sports, music, and hobbies. The questionnaire does not capture levels of physical activity outside of these recreational activities. Data were positively skewed with little gradation, hence for the current analyses, a binary variable was utilized which assessed any recreational activity involvement (yes/no). Additionally, five binary variables for highly endorsed recreational sports and hobbies were examined, including swimming, soccer, baseball, music, and art. See Fig. 1 for endorsement rates of all 29 activities assessed.

### Explanatory measures

#### Screen media activity (youth report)

Non-educational SMA was assessed by asking youth to indicate how long (none, < 30 min, 30 min, 1 h, 2 h, 3 h, or 4h hours) they were engaged in the following activities during weekdays and on the weekend: i) TV shows or movies; ii) videos; iii) video games on a computer, console, phone or other device; iv) messaging on a cell phone, tablet, or computer; v) social networking sites; and vi) video chat.

#### Psychopathology (parent report)

Youth externalizing and internalizing psychopathology syndrome t-scores from the Child Behavior Checklist were utilized in the analyses [22].

#### Cognition (youth performance)

The neurocognitive assessment included seven NIH Toolbox® tasks, the Rey Auditory Verbal Learning Test, and the Weschsler Intelligence Scale for Children [23].

#### Social environment (youth/parent report)

The social environment domain was assessed using the youth and parent-reported prosocial behavior subscale of Strengths and Difficulties Questionnaire, [24] the acceptance subscale of the Children’s Reports of Parental Behavior for parent and caregiver, [25] the Parent Monitoring Questionnaire, [26] and the conflict subscale of the Family Environment Scale [27].

#### Covariates

The following sociodemographic variables were included in all statistical models and were dummy coded: sex (M/F), race/ethnicity (White, Black, Hispanic, Asian, Other), parent education (<high school diploma, high school diploma or equivalent, college, Bachelor’s degree, Postgraduate degree), household income (< 50 K, 50-100 K, > 100 K), and marital status (single parent household, married/living together). Youth and parent age were included as continuous variables.

### Statistical analysis

#### Group comparisons

Initial groupwise comparisons on all explanatory measures were performed between youth who did (*n* = 8308) and not did (*n* = 946) engage in at least one recreational activity. Using R package ‘tableone’, one-way ANOVAs were conducted for continuous variables and chi-square tests were conducted for categorical variables.

#### Group factor analysis

Next, Group Factor Analysis (GFA) [28] was conducted to generate a set of explanatory factors that account for variance in SMA and other individual (i.e., cognition, psychopathology) and interpersonal (i.e., social environment) factors. GFA is an unsupervised learning technique that identifies latent variables across “groups” of variables. This technique allows identification of factors that selectively load onto a construct (e.g., SMA) or across constructs (e.g., SMA and psychopathology). It then uses these factors to determine whether the construct specifically is associated with outcomes of interest (e.g., recreational activity endorsement). For the current analyses, four variable groups were entered, including SMA, psychopathology, cognitive function, and social environment, using the ‘GFA’ R package. The solution comprises a set of group factors (GFs) which load onto the correlated groups of variables. GFA estimation was repeated 10 times with different seeds of randomized numbers in order to identify robust GFs which were consistent across sampling chains. The robust GFs were selected by two criteria. First, posterior means of GF components obtained from the ten sampling chains were required to pass a Pearson correlation threshold of 0·7 in order to be considered as the “same”. Second, a GF was deemed robust if it was identified at least 70% of the time across the 10 replicates. The robust GF scores were then averaged across the ten replicated analyses and utilized in the mixed model analyses.

#### Mixed models

Subsequent association analyses were conducted within a generalized linear mixed models (GLMMs) framework, using a logistic link to predict recreational activity involvement (R package: ‘glmmTMB’). Parameters of the mixed model were estimated by the Restricted Maximum Likelihood. Research site and siblings nested within site were entered as random intercepts. In the first pass, a GLMM analysis of a base model was conducted where sociodemographic variables (youth age, sex, and race/ethnicity, as well as parent age, education, marital status, household income) were entered as independent variables predicting involvement in any recreational activity (yes/no). In a second pass, a full model was conducted where the robust SMA, psychopathology, cognitive function, and social environment-related GFs were entered as additional independent variables, alongside the sociodemographic measures (youth age, sex, and race/ethnicity, as well as parent age, education, marital status, household income). Comparison between the base and full model was conducted using the ANOVA F-test and Bayesian Information Criterion (BIC). The nested models (i.e., base and full models) were then repeated for highly endorsed sports and hobbies, including soccer, music, swimming, baseball, and art in five separate models.

## Results

### Group comparison

Recreational activity endorsement was high, with 89·8% of parents endorsing youth involvement in at least one recreational activity, for whom the mean number of activities endorsed was 3·4 and the maximum number was 23 (Fig. 1). Highly endorsed sports and hobbies included soccer (40·2%), music (39·0%), swimming (31·5%), baseball (26·8%), and art (19·3%). Sociodemographic group differences between those who did and did not engage in at least one recreational activity are reported in Table 1. Group differences for SMA, cognition, psychopathology, and social environment measures are reported in Suppl. Table 1. In unadjusted groupwise comparisons, youth who engaged in recreational activities spent less time engaging with SMA (all *p*s < .001), exhibited lower total psychopathology symptoms (*p* < .001), performed better on cognitive tasks (all *p*s < .001), experienced less family conflict (*p* < .001), greater parental acceptance (*p* < .001) and monitoring (*p* < .001), and exhibited greater prosocial behavior (*p* = .007) than youth who did not engage in recreational activities.

**Table 1:** Sociodemographic data on youth from the ABCD cohort

	No activity endorsement *N* = 946	Activity endorsement *N* = 8308	*p*
Age (mean [SD])	9.8 (0.6)	9.9 (0.6)	<.001
Male (%)	477 (50.4)	4332 (52.1)	.33
Race/Ethnicity (%)			<.001
White	281 (29.7)	4925 (59.3)	
Black	296 (31.3)	891 (10.7)	
Hispanic	248 (26.2)	1461 (17.6)	
Asian	13 (1.4)	179 (2.2)	
Other	108 (11.4)	852 (10.3)	
Household income (%)			<.001
< $50 K	611 (64.6)	1899 (22.9)	
$50-100 K	243 (25.7)	2406 (29.0)	
> $100 K	92 (9.7)	4003 (48.2)	
Parent education (%)			<.001
≤ HS	336 (35.5)	719 (8.6)	
College	392 (41.4)	1901 (22.9)	
≥ Bachelor’s degree	218 (23.0)	5688 (68.5)	
Married/live together (%)	561 (59.3)	6609 (79.5)	<.001
Parent Age (mean [SD])	37.2 (7.1)	40.5 (6.6)	<.001

### Group factor analysis

**Fig. 2:**
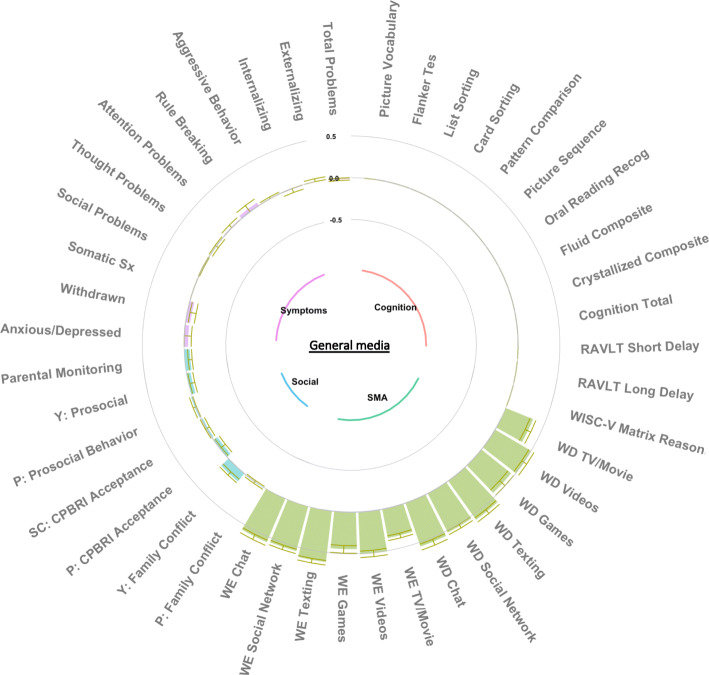
General media group factor

**Fig. 3:**
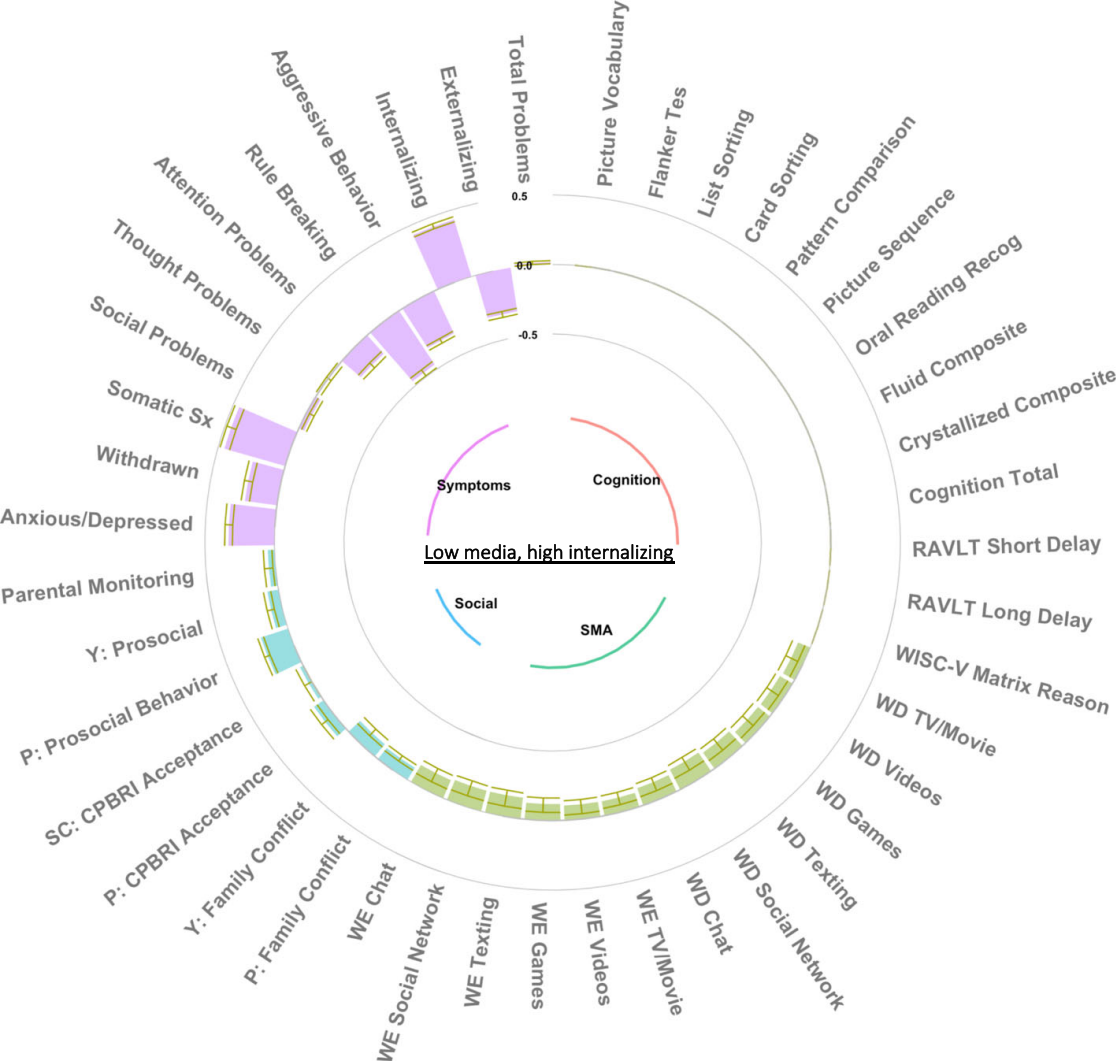
Low media, high internalizing symptoms group factor

**Fig. 4:**
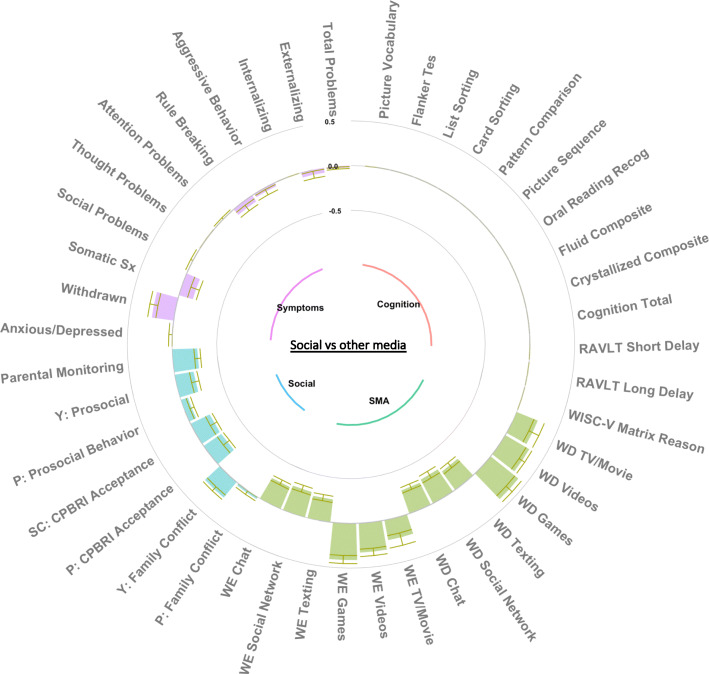
Low social media, high other media (movies, videos, games) group factor

The GFA procedure yielded 15 robust GFs which occurred in at least 70% of the ten replicated analyses and passed the Pearson correlation threshold of 0·7. These GFs explained 33·7% of the total variance (suppl. Fig. 1), including 42·7% of SMA, 44·5% of cognitive function, 13·7% of psychopathology, and 11·6% of social environment variance (suppl. Fig. 2). Five GFs were strictly SMA-related, one was cognitive-related, two were psychopathology-related, five were related to the social environment, one was psychopathology- and SMA-related, and one was psychopathology- and cognitive-related. These GFs were largely orthogonal (suppl. Fig. 3). All 15 GFs were extracted for GLMM analysis and Figs. 2, 3, and 4 depict the three SMA-related GFs that showed some association with recreational activity involvement, while all other GF figures are available in the supplement (suppl. Figs. 4, 5, 6, 7, 8, 9, 10, 11, 12, 13, 14 and 15).

### Mixed model findings

#### Engagement with any recreational activity

**Fig. 5:**
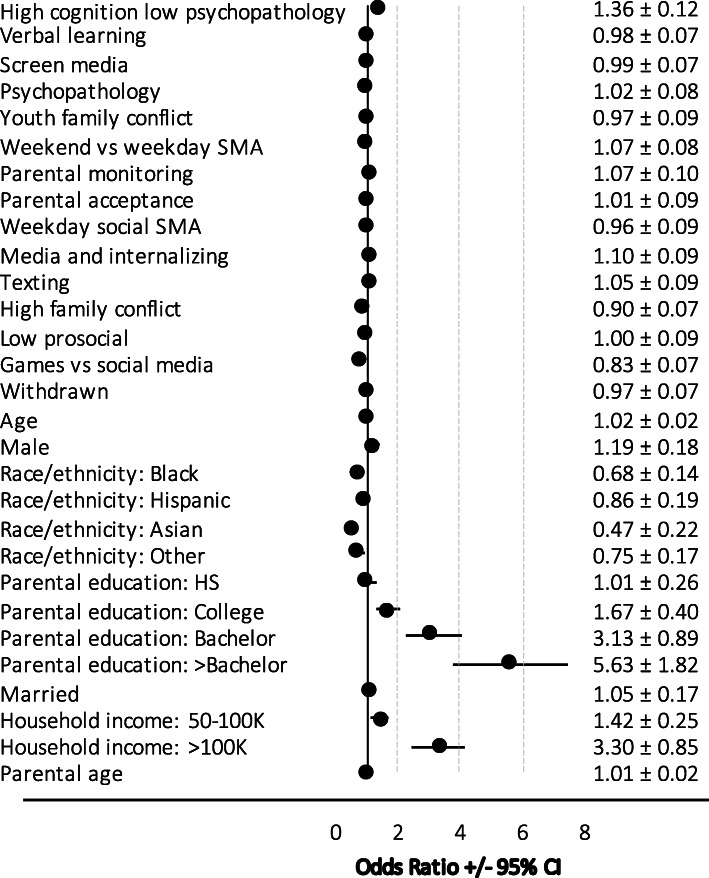
Factors associated with any recreational activity endorsement (of 29 activities assessed)

GLMM analysis showed that the full model (including 15 SMA, cognitive function, social environment, and psychopathology GFs and sociodemographic independent variables) for predicting involvement in any recreational activity (*R*^*2*^ = 0·35) did not significantly improve the base model which comprised of age, sex, race/ethnicity, parental education, marital status, household income, and parent age (*R*^*2*^ = 0·34, *BIC =* 5146·9, ∆*BIC* = 52·8, *LRT* = 84·2) (Fig. 5).

Of the six SMA-related GFs, two were significantly associated with recreational activity endorsement when adjusting for sociodemographic, individual, and interpersonal factors in the full model (Fig. 5). For every increase in one standard deviation (SD) on the ‘high games/movies and low social media’ GF, youth were 0·83 (*95% CI* 0·76–0·89) times as likely to endorse recreational engagement. For every one SD increase on the ‘low SMA and high internalizing psychopathology’ GF, youth were 1·10 (1·02–1·18) times more likely to endorse recreational engagement.

In terms of sociodemographic factors, compared to White youth, Black, Asian, and other race/ethnicity youth were 0·68 (*95% CI* 0·54–0·85), 0·47 (0·25–0·87), and 0·75 (0·58–0·97) times as likely to endorse any activity involvement, respectively. Compared to youth where parents did not complete high school, youth of college attendees, Bachelor, or > Bachelor graduates were 1·67 (1·26–2·22), 3·13 (2·24–4·37), and 5·63 (3·83–8·29) times more likely to endorse recreational activity engagement, respectively. Similarly, youth from middle ($50-100 K) and higher (>$100 K) income households were 1·42 (1·16–1·72) and 3·30 (2·47–4·42) times more likely to endorse engagement than youth from lower (<$50 K) income households, respectively.

#### Engagement with highly endorsed sports and hobbies

The five full models (including 15 SMA, cognitive function, social environment, and psychopathology GFs and sociodemographic independent variables) for predicting highly endorsed recreational activities, including soccer (*R*^*2*^ = 0·20), swimming (*R*^*2*^ = 0·11), baseball (*R*^*2*^ = 0·24), music (*R*^*2*^ = 0·23), and art (*R*^*2*^ = 0·09) did not significantly improve base models which comprised of age, sex, race/ethnicity, parental education, marital status, household income, and parent age (soccer [*R*^*2*^ = 0·20, *BIC* = 11,394·1, ∆*BIC* = 102·4, *LRT* = 34·6], swimming [*R*^*2*^ = 0·10, *BIC* = 11,153·5, ∆*BIC* = 90·0, *LRT* = 47·0], baseball [*R*^*2*^ = 0·23, *BIC* = 9792·8, ∆*BIC* = 100·1, *LRT* = 36·8], music [*R*^*2*^ = 0·21, *BIC* = 11,142·5, ∆*BIC* = 64·1, *LRT* = 201·0], art [*R*^*2*^ = 0·08, *BIC* = 8978·9, ∆*BIC* = 86·0, *LRT* = 51·0]) (see Suppl. Figs. 16, 17, 18, 19 and 20).

Of the six SMA-related GFs, three were significantly associated with various sports and hobbies when adjusting for sociodemographic, individual, and interpersonal factors in full models (Suppl. Figs. 16, 17, 18, 19 and 20). For every one SD increase on the ‘high games/movies and low social media’ GF, youth were less likely to engage in swimming (*adjusted OR* = 0·91, *95% CI* 0·85–0·96), soccer (0·91, 0·86–0·96), baseball (0·91, 0·85–0·97), and music (0·88, 0·83–0·94). For every one SD increase on the ‘high general SMA’ GF, youth were less likely to endorse soccer (0·93, 0·88–0·98) and baseball (0·92, 0·86–0·98). For every one SD increase on the ‘low SMA use and high internalizing psychopathology’ GF, youth were more likely to endorse swimming (1·07, 1·02–1·13) and art (1·09, 1·03–1·16), and less likely to endorse baseball (0·92, 0·87–0·97).

For each activity, findings related to parent education and household incomes were mostly consistent with the overall activity model described above. Compared to females, males were less likely to endorse swimming (*adjusted OR* = 0·87, *95% CI* = 0·79–0·95), music (0·66, 0·60–0·72), and art activities (0·45, 0·40–0·51). In contrast, males were 1·96 (1·78–2·16) and 3·57 (3·20–3·99) times more likely than females to endorse soccer and baseball involvement, respectively. Compared to White youth, Black youth were less likely to endorse soccer (0·39, 0·32–0·47) and baseball (0·37, 0·30–0·46), Asian youth were more likely to endorse swimming (1·44, 1·06–1·95) and music (1·60, 1·15–2·23), and less likely to endorse soccer (0·36, 0·26–0·51) and baseball (0·23, 0·14–0·36), and Hispanic youth were less likely to endorse baseball (0·76, 0·65–0·91).

## Discussion

This study used a large dataset of 9–10-year-old youth to isolate the relationship between screen media activity and youth recreational activity involvement, when accounting for other sociodemographic, cognitive, psychopathology, and social environment factors. Overall, GF-augmented models did not provide a significantly better fit to the data than base models, indicating that sociodemographic factors, particularly socio-economic status, explain more variance in rates of recreational activity engagement than other factors, such as SMA. While greater SMA was related to activity displacement in unadjusted group comparisons, most forms of SMA were no longer significantly associated with recreational activity engagement when accounting for confounding factors. The SMA effects that were observed in adjusted models were small, showing only marginal associations with some activities. Taken together, and contrary to the displacement hypothesis, this study did not find strong evidence that non-educational SMA was at the expense of other recreational activity engagement in 9–10-year-old youth, when accounting for other individual, interpersonal, and sociodemographic factors.

The current findings are in agreement with some previous research which shows SMA does not compete with other activities [4, 16, 17] and is in disagreement with other studies which conclude SMA displaces physical and outdoor activities in youth and adolescence [10–13]. Consistent with other data, exploration of different types of recreational activities and different forms of media show that where relations do exist, they are nuanced [14, 15]. For example, the current study provided some indication that “technoactive” (i.e., high social SMA, high recreational engagement) and “socially isolated SMA” (i.e., high general SMA, low group-based recreational engagement) clusters of youth exist. Although, prior studies of adolescents have identified stronger associations [11, 14, 15, 18, 19]. These inconsistencies may be due to the relatively early developmental period under study and suggest that patterns of behavior may continue to diverge throughout adolescence.

There are several noteworthy aspects of the current study which may account for some of the observed differences. Firstly, this is the first large-scale study of a preadolescent population and the impact of SMA on youth recreational activity involvement may change as a function of age. Accordingly, stronger associations between high SMA and low physical activity engagement have been previously observed in older adolescents [16]. To date, most studies in this field have reported on cross-sectional data. Longitudinal analysis of this large cohort will provide further clarification on possible clusters of youth and the relative interdependence of SMA use and recreational activity involvement throughout adolescence. Secondly, studies examining associations between SMA and other outcome variables are complicated by the fact that these activities strongly correlate with other factors, such as sociodemographic characteristics [29]. Using a mixed model analytic approach, the current study demonstrated that associations between SMA, sports, and other hobbies are minimal when confounding factors are appropriately taken into account.

Further to this point, and consistent with other data, the most robust finding from the current study was that youth from higher socio-economic families were more likely to engage in recreational activities than youth from lower socio-economic backgrounds [30, 31]. Previous studies have demonstrated that lower socio-economic status and high-minority areas have reduced access to recreational activity facilities, bike trails, gym equipment, and perceived safe outdoor spaces [32]. Similarly, associations between poverty and recreational inactivity have been observed across the life span [32]. Therefore, greater availability of free recreational resources and programs could be beneficial to families with limited resources. Of note, many of the recreational activities examined in the current study require some form of registration and paid membership. Associations between socio-economic status and endorsement of free leisure activities may differ to those observed here. Further exploratory work examining causes of non-participation is warranted.

Key strengths of this study include utilization of data-driven techniques (i.e., GFA) to distinguish clusters of youth who share similar patterns of behavior or characteristics. Identifying unique patterns of SMA engagement, cognition, social environment, and psychopathology allowed for complex patterns of behavior to be adequately characterized. Furthermore, using a mixed model analytic approach allowed for appropriate adjustment of the complexity of factors that influence youth behaviors. This provided more robust conclusions than reported in some previous association studies. This study also has several limitations. First, this is a cross-sectional assessment, which enabled establishment of associations but does not address causation or directionality. The longitudinal component of ABCD will be essential to begin to delineate causal pathways. Second, unmeasured confounding factors may be contributing to the observed associations. Third, the initial ABCD assessments of media activity are limited to self-report, which may introduce a number of biases and could be improved by more direct assessments of SMA. Fourth, recreational activity involvement was examined as a binary outcome variable due to positively skewed data with little gradation. Therefore, associations between SMA and other factors on the level of activity involvement could not be explored. Fifth, the ABCD cohort are a probability sample which is not necessarily representative of the US population. Finally, the present study was limited to examination of youth aged 9–10 years, which inhibited exploration of age as a moderating factor between SMA and recreational activity displacement. Although, it should be noted that examination of this younger cohort is unique to the existing evidence base, where previous studies have focused on associations in adolescents.

## Conclusion

This study found that screen media activity does not appear to largely displace engagement with other recreational activities, including sports and hobbies, in preadolescent youth. Where associations between SMA and other activities were observed, effects were nuanced and small at best. Considering the recent shift in leisure time spent on non-educational SMA in childhood, it is encouraging that SMA does not appear to impede engagement with sports and hobbies, which are potentially more beneficial for health and cognitive development [9]. Importantly, the findings attribute much of the variance in recreational activity endorsement to socio-economic factors. Longitudinal analysis of this cohort will provide clarification on whether particular forms of screen media more greatly impacts engagement with other activities when youth enter adolescence.

## Supplementary Information

**Additional file 1: Supplement Materials.** Additional results are provided.

**Additional file 2: STROBE Statement – Checklist** of items that should be included in reports of cross-sectional studies. The STROBE checklist has been used in conjunction with this article. Page numbers of the manuscript are provided for relevant criteria/details.

## Data Availability

The datasets generated and/or analysed during the current study are available in the National Institute of Mental Health Data Archive repository, https://nda.nih.gov/abcd. Data used in the preparation of this article were obtained from the Adolescent Brain Cognitive Development (ABCD) Study (https://abcdstudy.org), held in the NIMH Data Archive (NDA). This is a multisite, longitudinal study designed to recruit more than 10,000 children age 9–10 and follow them over 10 years into early adulthood. A listing of participating sites and a complete listing of the study investigators can be found at https://abcdstudy.org/scientists/workgroups/. The ABCD data repository grows and changes over time. The ABCD data used in this report came from 10.15154/1504431 (DOI). DOIs can be found at https://nda.nih.gov/study.html?id=796.

## Abbreviations

ABCD: Adolescent Brain Cognitive Development
BIC: Bayesian Information Criterion
GF: Group factor
GFA: Group factor analysis
GLMM: Generalized linear mixed models
SMA: Screen media activity
