# Supplementary material for: Screen media activity does not displace other recreational activities among 9–10 year-old youth: a cross-sectional ABCD study®
Source: BMC Public Health. 2020 Nov 25;20:1783. doi: 10.1186/s12889-020-09894-w (PMC7687784; doi:10.1186/s12889-020-09894-w)
Supplement: Supplementary file 1 — Additional file 1: Supplement Materials. Additional results are provided. [file 12889_2020_9894_MOESM1_ESM.docx]

**Supplement materials**

**Suppl. Table 1: Group comparisons on explanatory measures. (n=9,254)**

|  | **No activity endorsement**  N=946 | **Activity endorsement**  N=8,308 | ***p*** |
| --- | --- | --- | --- |
|  | **Mean (SD)** | **Mean (SD)** |  |
| **SMA** |  |  |  |
| *Weekdays (hours)* | | | |
| TV/Movies | 1.41 (1.27) | 1.05 (1.06) | <.001 |
| Videos | 1.25 (1.34) | 0.83 (1.10) | <.001 |
| Games | 1.22 (1.33) | 0.86 (1.08) | <.001 |
| Texting | 0.29 (0.71) | 0.19 (0.50) | <.001 |
| Social Networks | 0.18 (0.58) | 0.09 (0.37) | <.001 |
| Video Chat | 0.24 (0.60) | 0.16 (0.43) | <.001 |
| *Weekends (hours)* | | | |
| TV/Movies | 1.82 (1.42) | 1.58 (1.23) | <.001 |
| Videos | 1.54 (1.47) | 1.07 (1.28) | <.001 |
| Games | 0.31 (0.76) | 1.18 (1.27) | <.001 |
| Texting | 0.31 (0.76) | 0.23 (0.59) | <.001 |
| Social Networks | 0.19 (0.64) | 0.11 (0.46) | <.001 |
| Video Chat | 0.30 (0.76) | 0.20 (0.56) | <.001 |
| **Psychopathology** |  |  |  |
| Anxious/depressed | 53.45 (6.10) | 53.46 (5.90) | .97 |
| Withdrawn | 54.38 (6.67) | 53.21 (5.45) | <.001 |
| Somatic symptoms | 54.72 (6.09) | 54.81 (5.93) | .67 |
| Social problems | 53.75 (5.78) | 52.53 (4.43) | <.001 |
| Thought problems | 54.34 (6.70) | 53.69 (5.73) | .001 |
| Attention problems | 54.88 (7.13) | 53.65 (5.91) | <.001 |
| Rule breaking | 53.92 (5.96) | 52.47 (4.58) | <.001 |
| Aggressive behavior | 53.79 (6.81) | 52.59 (5.27) | <.001 |
| Internalizing | 48.50 (11.20) | 48.32 (10.40) | .62 |
| Externalizing | 47.34 (11.44) | 45.21 (10.05) | <.001 |
| Total problems | 47.03 (12.47) | 45.49 (10.95) | <.001 |
| **Cognition** |  |  |  |
| Picture vocabulary | 98.38 (15.29) | 108.95 (16.80) | <.001 |
| Flanker test | 92.44 (13.53) | 96.18 (13.45) | <.001 |
| List sorting | 94.58 (14.26) | 102.13 (14.48) | <.001 |
| Card sorting | 91.92 (12.74) | 97.89 (15.37) | <.001 |
| Pattern comparison | 89.42 (22.25) | 94.67 (21.77) | <.001 |
| Picture sequence | 96.89 (14.06) | 102.18 (16.10) | <.001 |
| Oral reading recognition | 95.57 (18.30) | 104.24 (19.03) | <.001 |
| Fluid composite | 88.41 (16.21) | 97.40 (17.08) | <.001 |
| Crystallized composite | 95.58 (16.85) | 107.84 (18.06) | <.001 |
| Cognition total | 90.69 (16.47) | 102.87 (17.45) | <.001 |
| Short Delay | 8.62 (3.23) | 9.89 (2.94) | <.001 |
| Long Delay | 8.08 (3.26) | 9.46 (3.09) | <.001 |
| Matrix Reasoning | 8.80 (2.89) | 10.18 (2.91) | <.001 |
| **Social environment** |  |  |  |
| P: Family conflict | 2.78 (2.08) | 2.49 (1.94) | <.001 |
| Y: Family conflict | 2.51 (2.08) | 1.93 (1.90) | <.001 |
| P: Acceptance | 2.75 (0.33) | 2.79 (0.29) | <.001 |
| Y: Acceptance | 2.68 (0.41) | 2.69 (0.38) | .35 |
| P: Prosocial behavior | 1.72 (0.45) | 1.76 (0.39) | .007 |
| Y: Prosocial behavior | 1.69 (0.38) | 1.68 (0.36) | .62 |
| Y: Parental monitoring | 4.28 (0.58) | 4.41 (0.48) | <.001 |

P=parent; Y=youth.

**Suppl Figure 1: The group factor analysis yielded 15 robust components/group factors. A shows the relative strength of loadings. B summarizes the percent of total variance explained by each group factor. In total, the 15 factors explained 33.7 +/- 4.5% variance of all variables.**

COG = cognitive function, CBCL = psychopathology, SOC = social environment, SMA = screen media activity.

**A.**

**
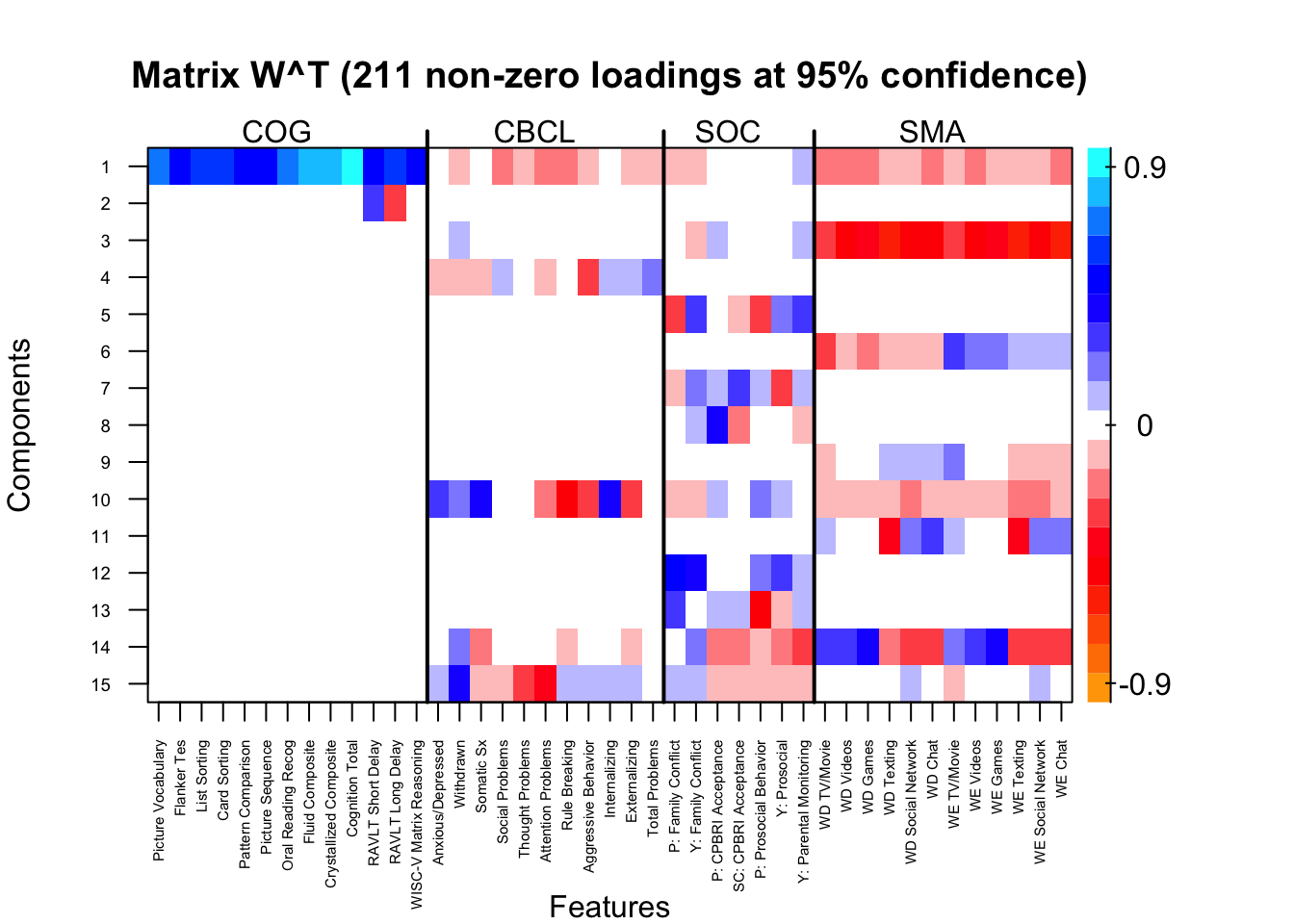
**

**B.**

**
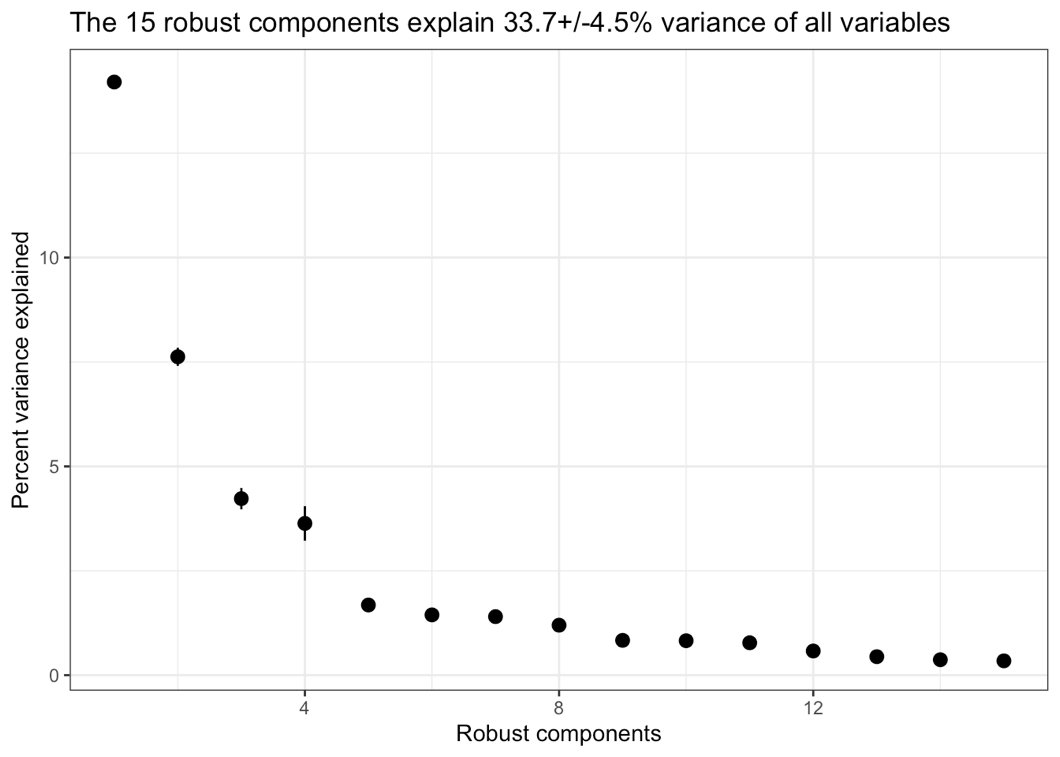
**

**Suppl Figure 2: Summary of the percent of cognitive, psychopathological, social environment, and screen media activity variance explained by each robust group factor. In summary, the 15 components explained 44.5 +/- 0.9% of variance in cognitive function (COG), 13.7 +/- 4.6% of psychopathology (CBCL), 29.8 +/- 11.6% of social environment (SOC), 42.7 +/- 8.1% of variance in screen media activity (SMA).**


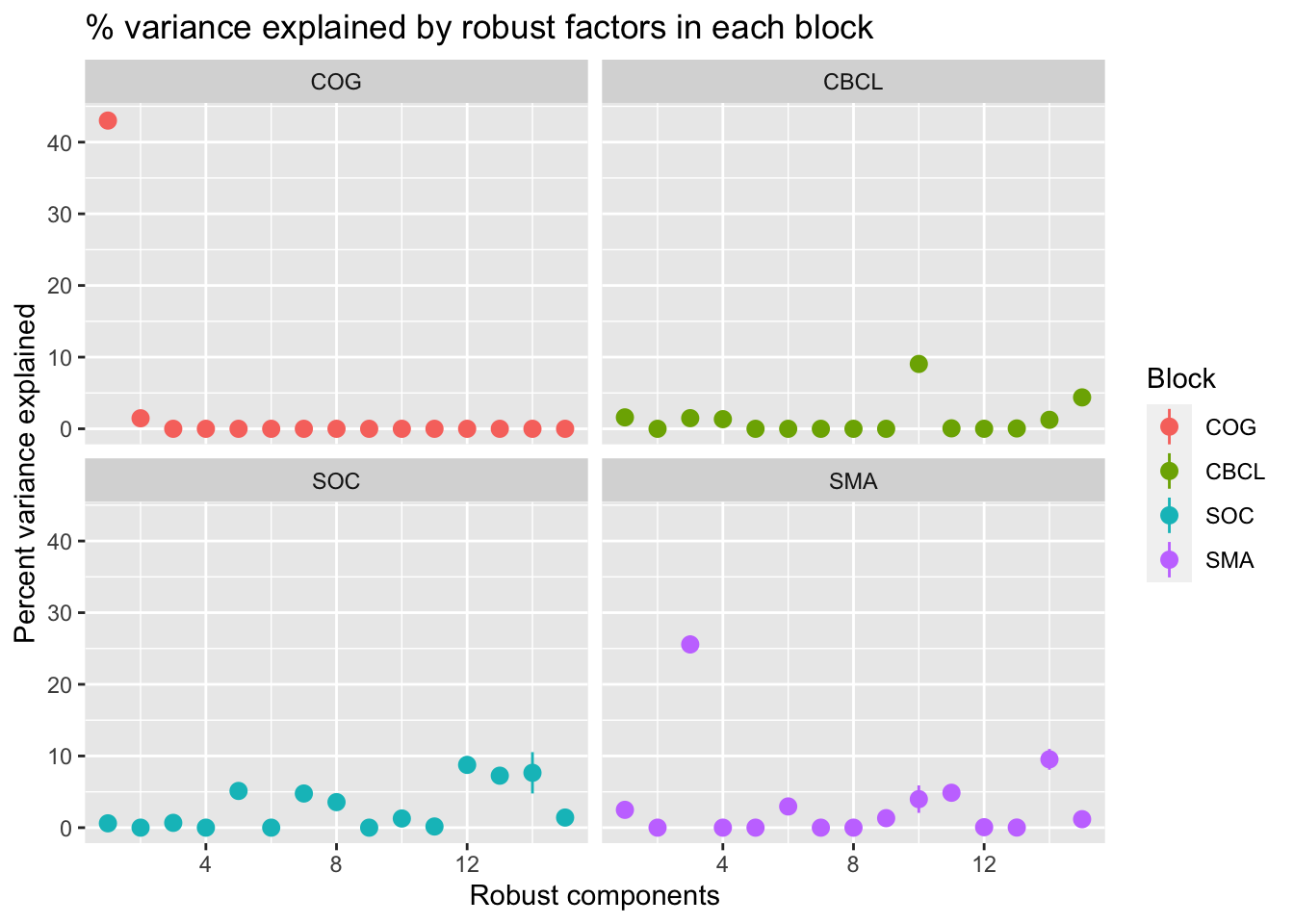


**Suppl Figure 3: Correlation matrix of group factors.**


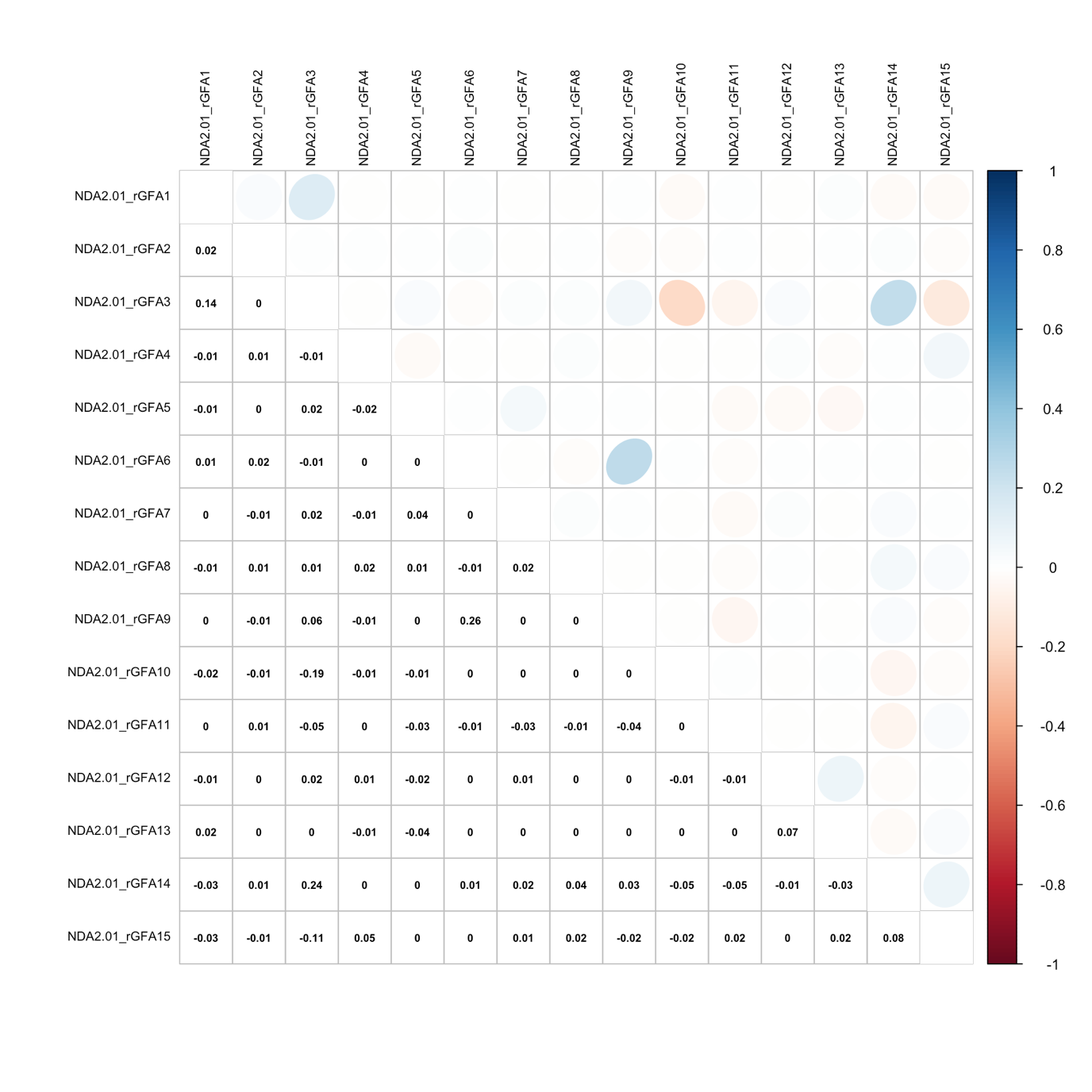


**Suppl Figure 4: Group factor analysis for high cognition, low psychopathology (GF 1)**

**
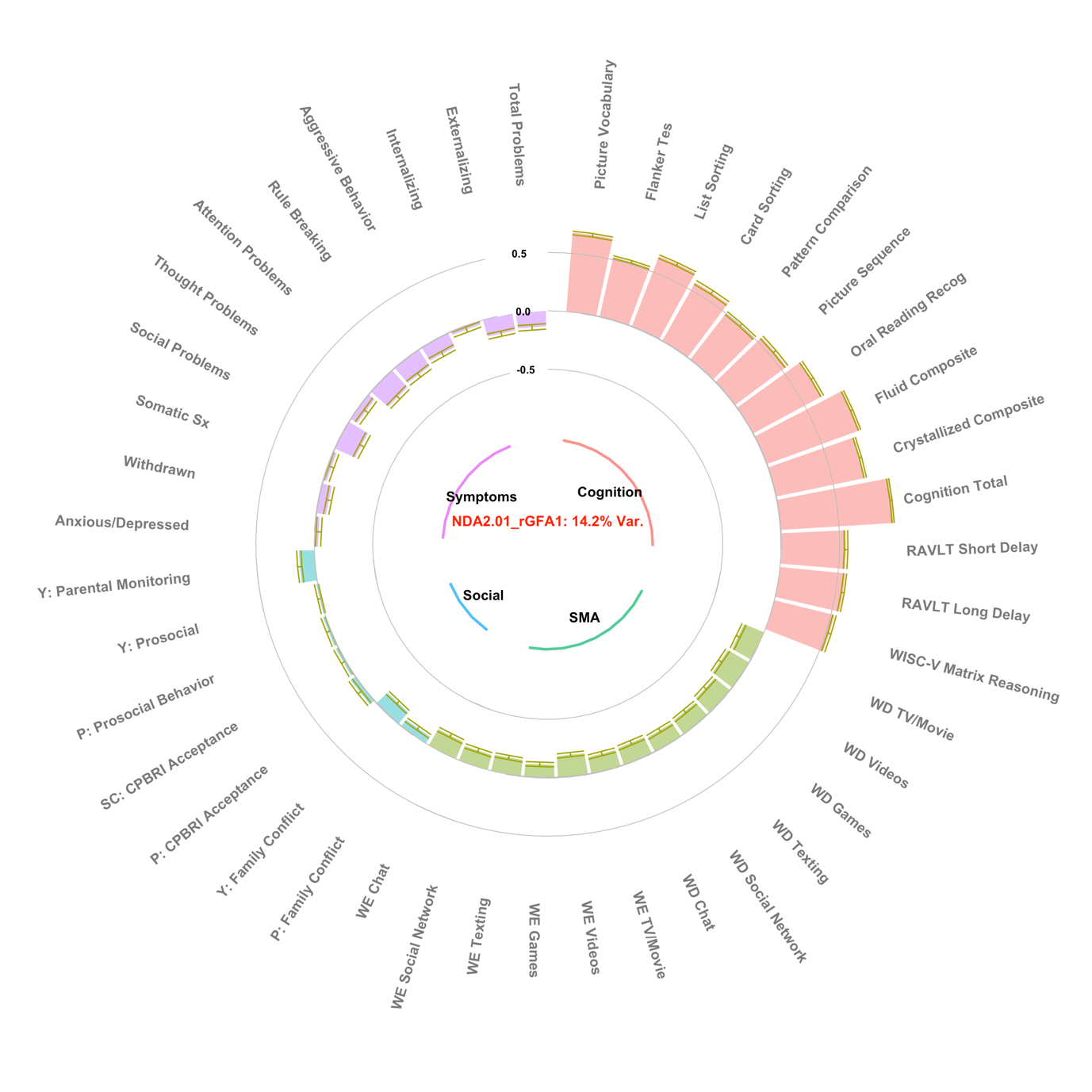
**

**Suppl Figure 5: Group factor analysis for verbal learning (GF 2)**

**
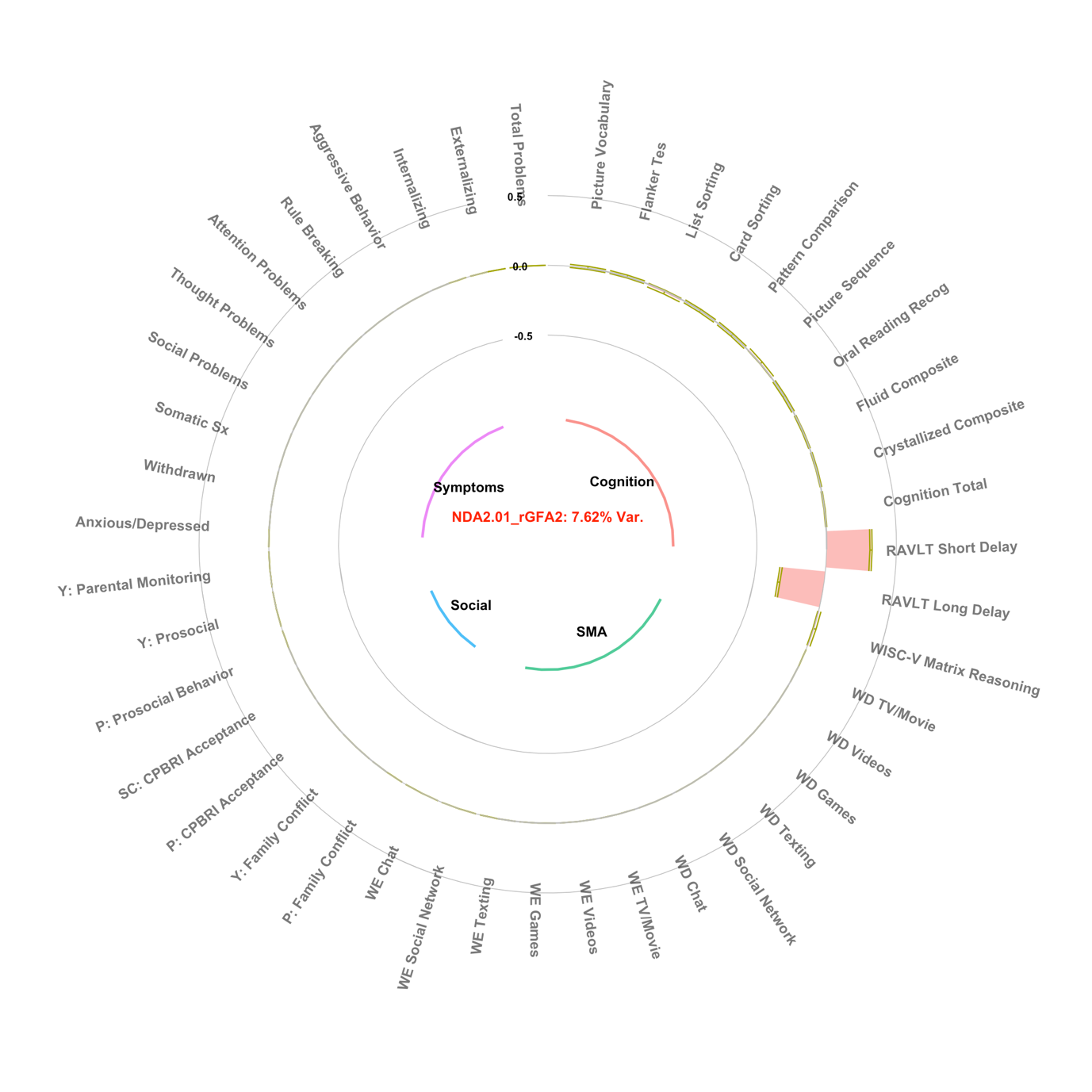
**

**Suppl Figure 6: Group factor analysis for psychopathology (GF 4)**

**
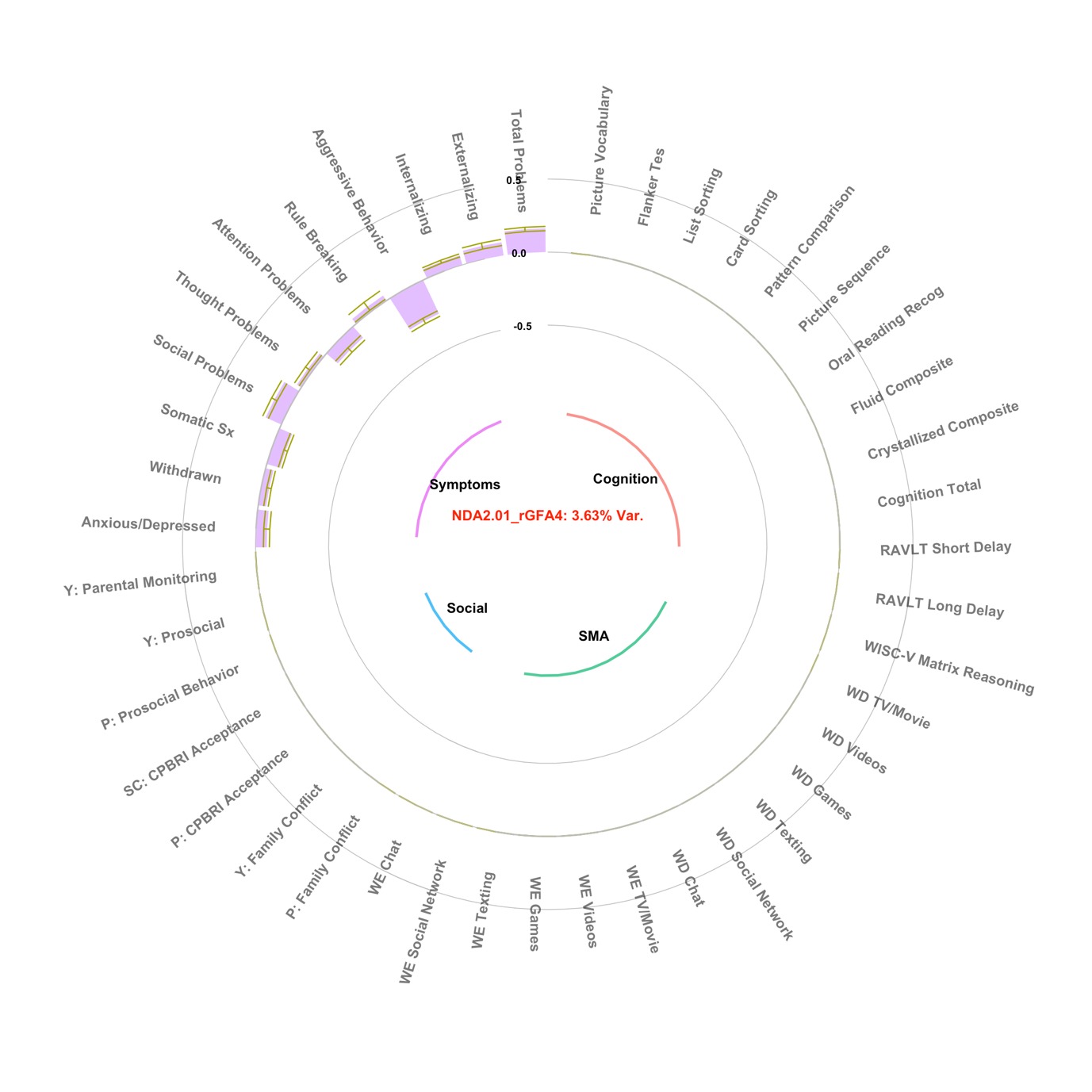
**

**Suppl Figure 7: Group factor analysis for youth family conflict (GF 5)**

**
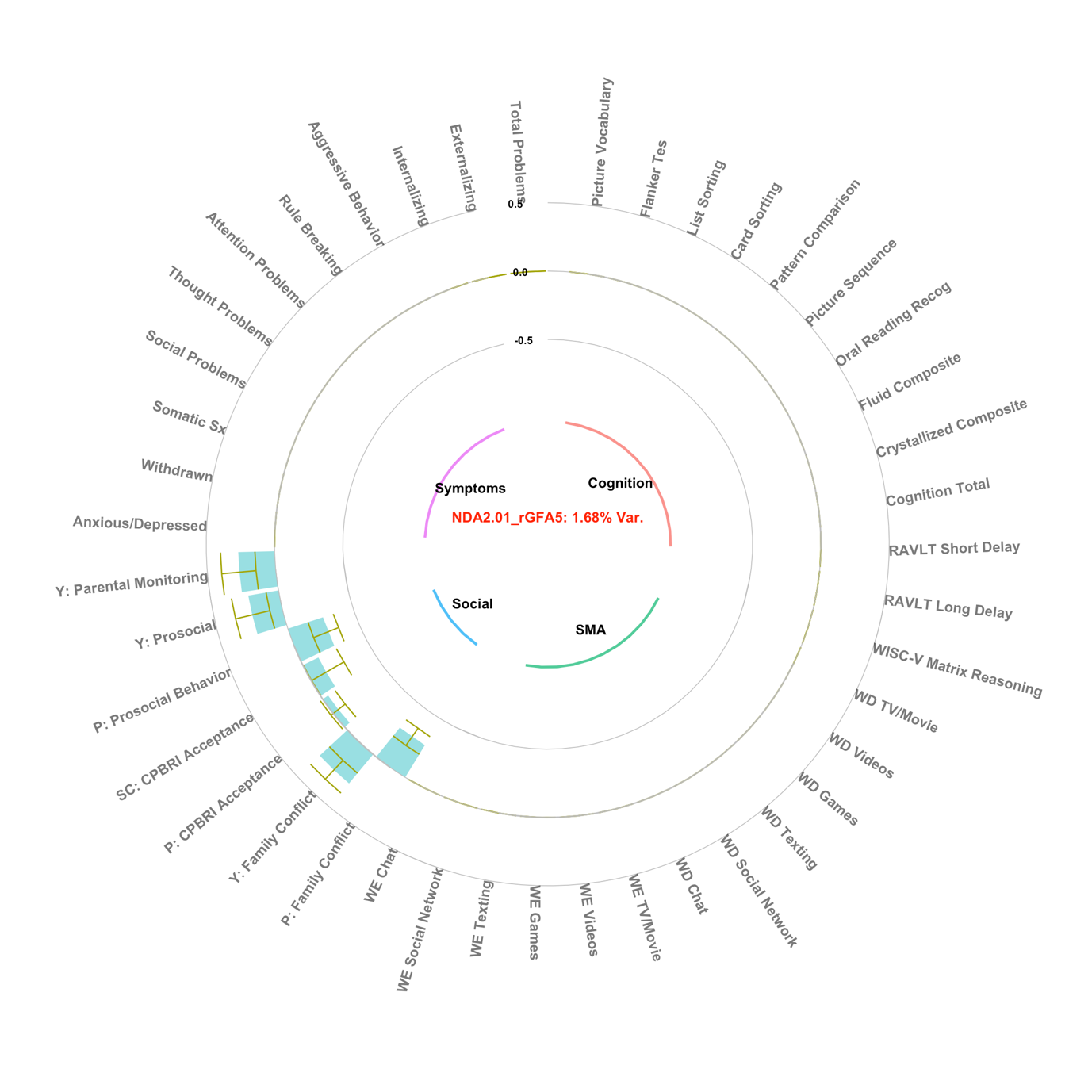
**

**Suppl Figure 8: Group factor analysis for weekend vs weekday screen media (GF 6)**

**
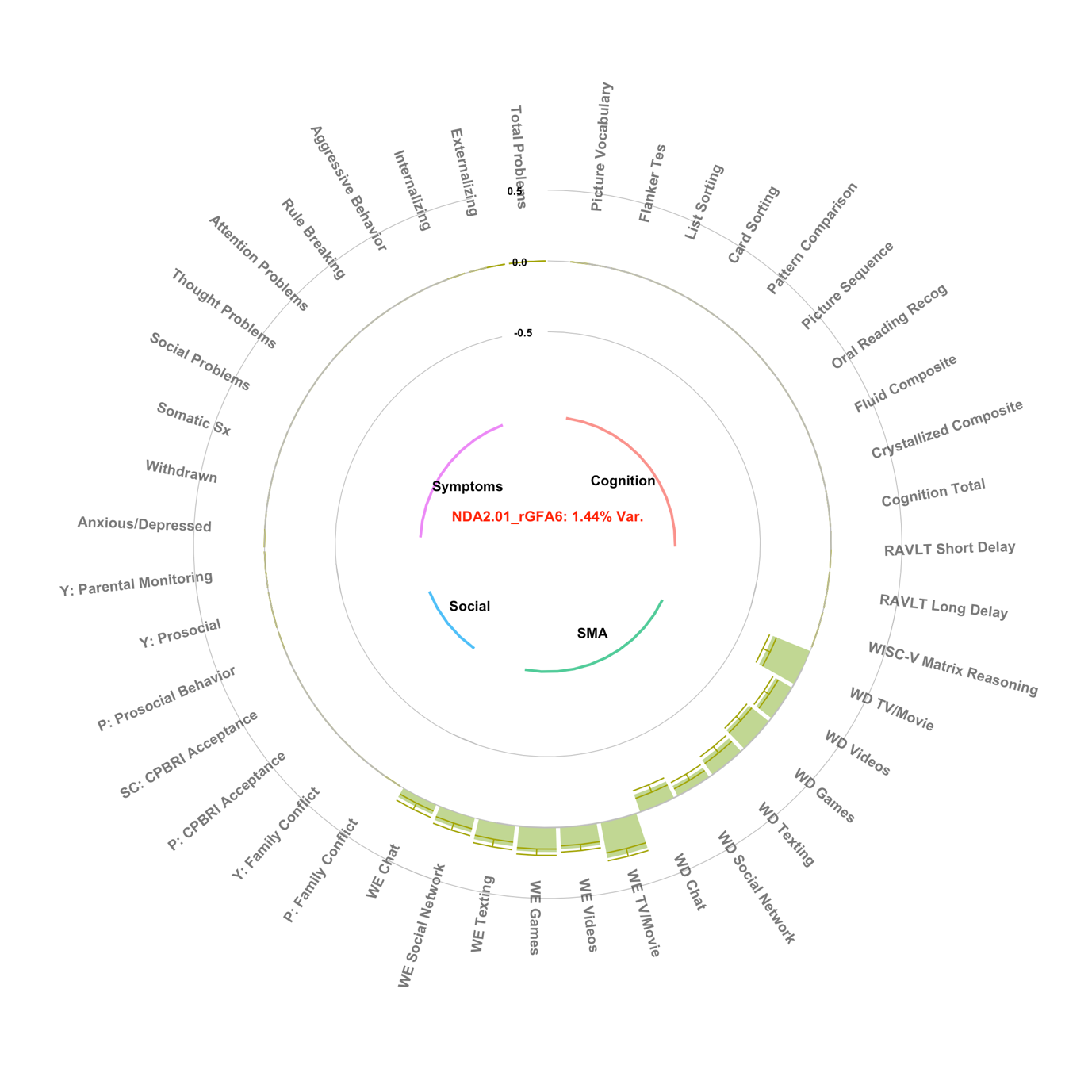
**

**Suppl Figure 9: Group factor analysis for parental monitoring (GF 7)**

**
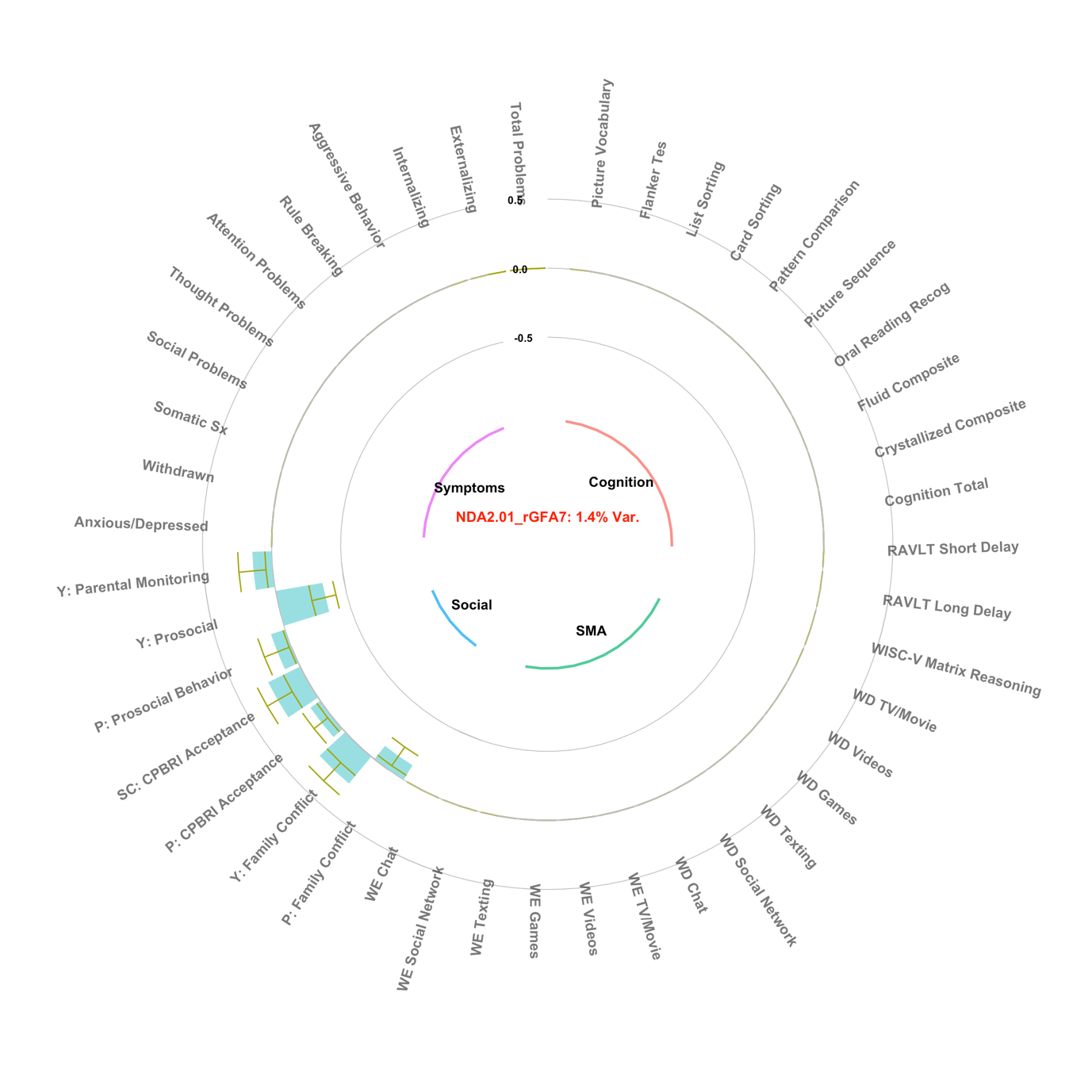
**

**Suppl Figure 10: Group factor analysis for parental acceptance (GF 8)**

**
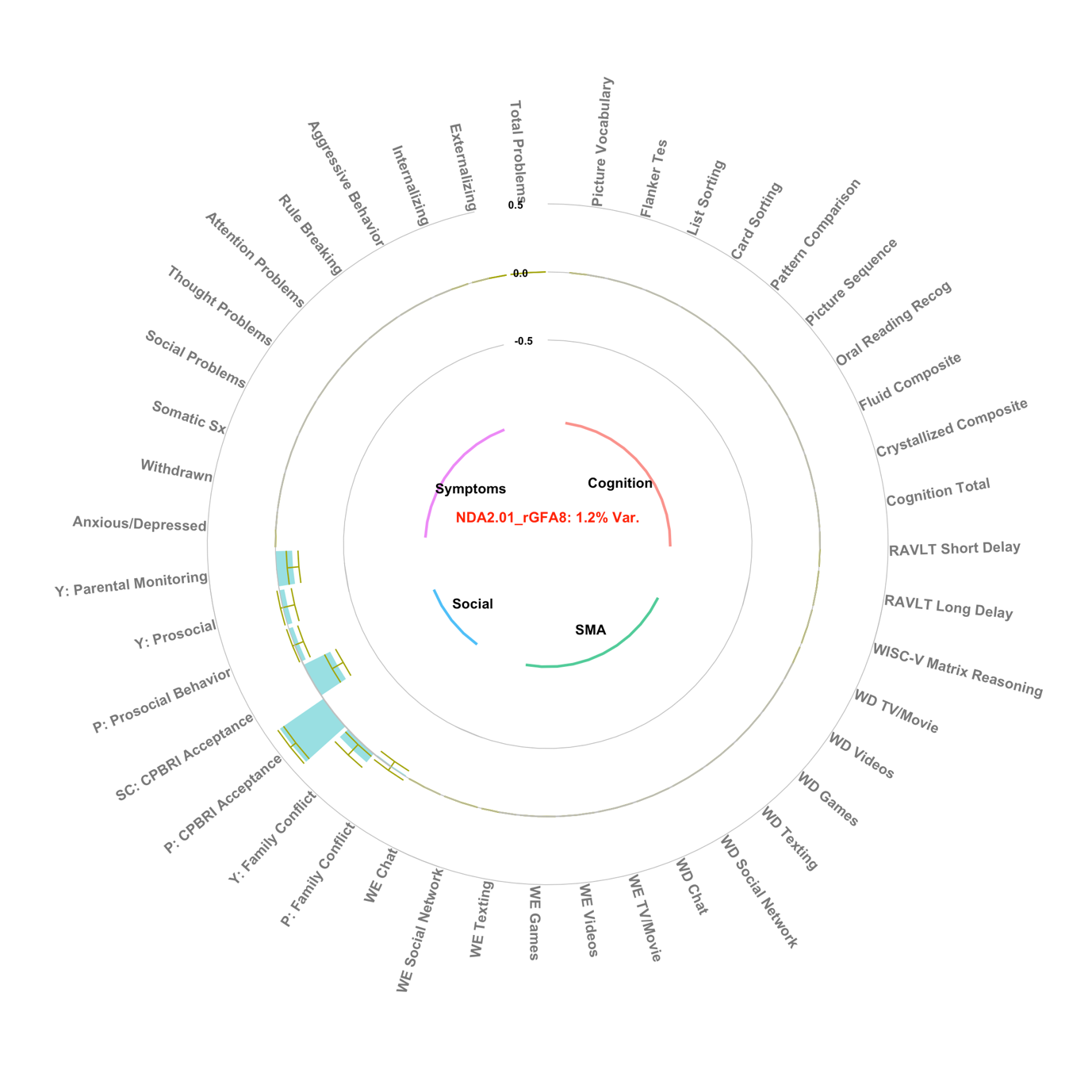
**

**Suppl Figure 11: Group factor analysis for weekday social media activity (GF 9)**

**
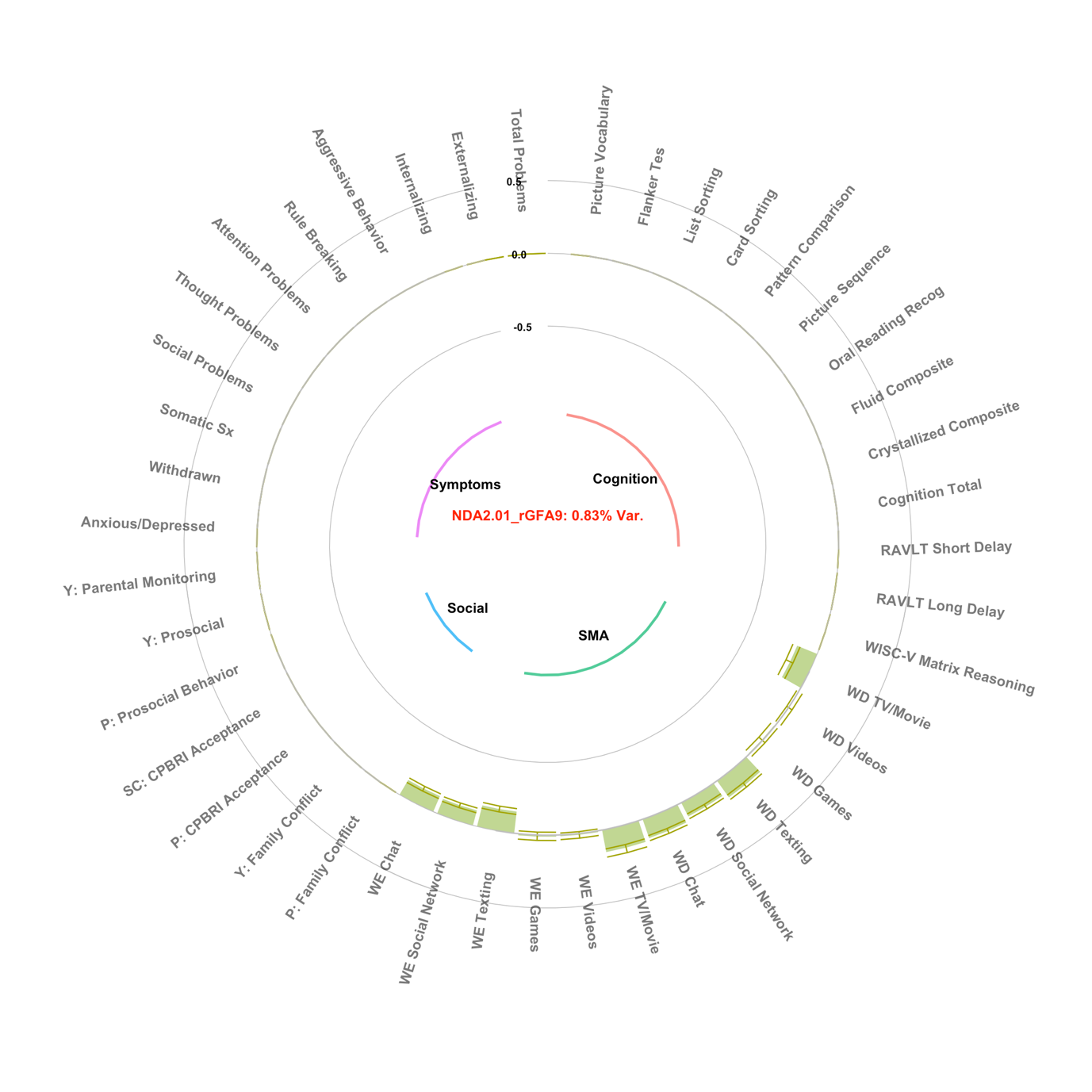
**

**Suppl Figure 12: Group factor analysis for texting (GF 11)**

**
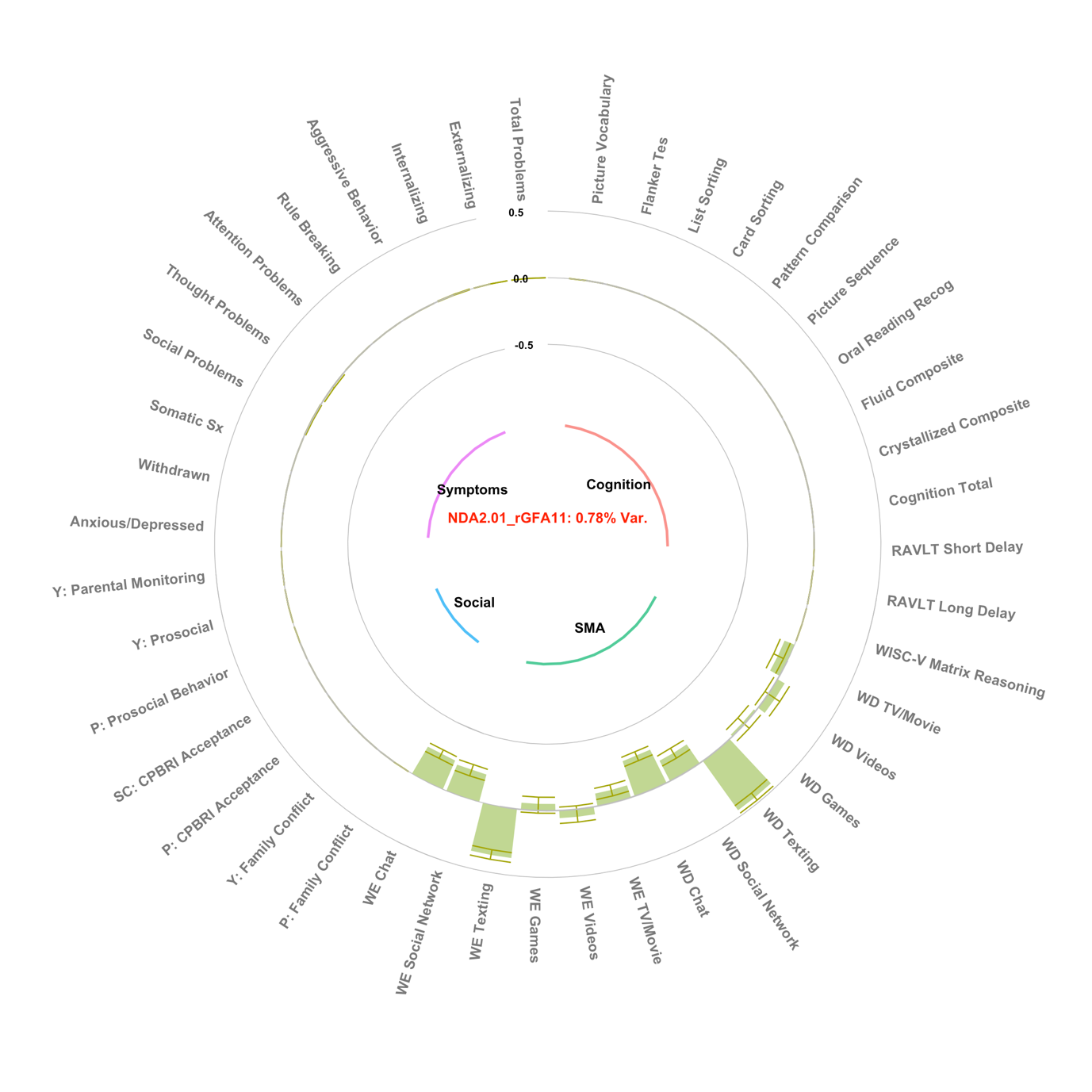
**

**Suppl Figure 13: Group factor analysis for high family conflict (GF 12)**

**
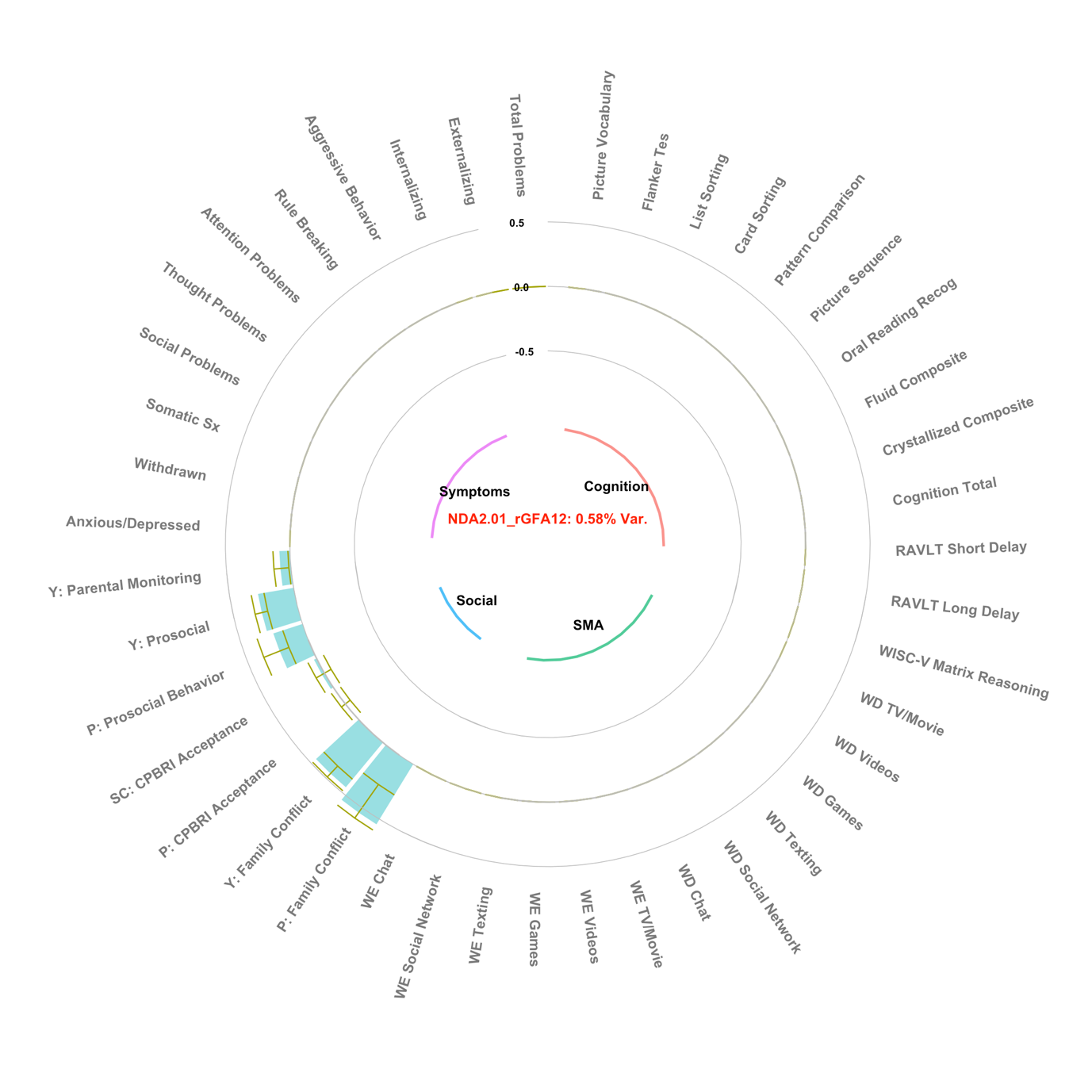
**

**Suppl Figure 14: Group factor analysis for low prosocial behavior (GF 13)
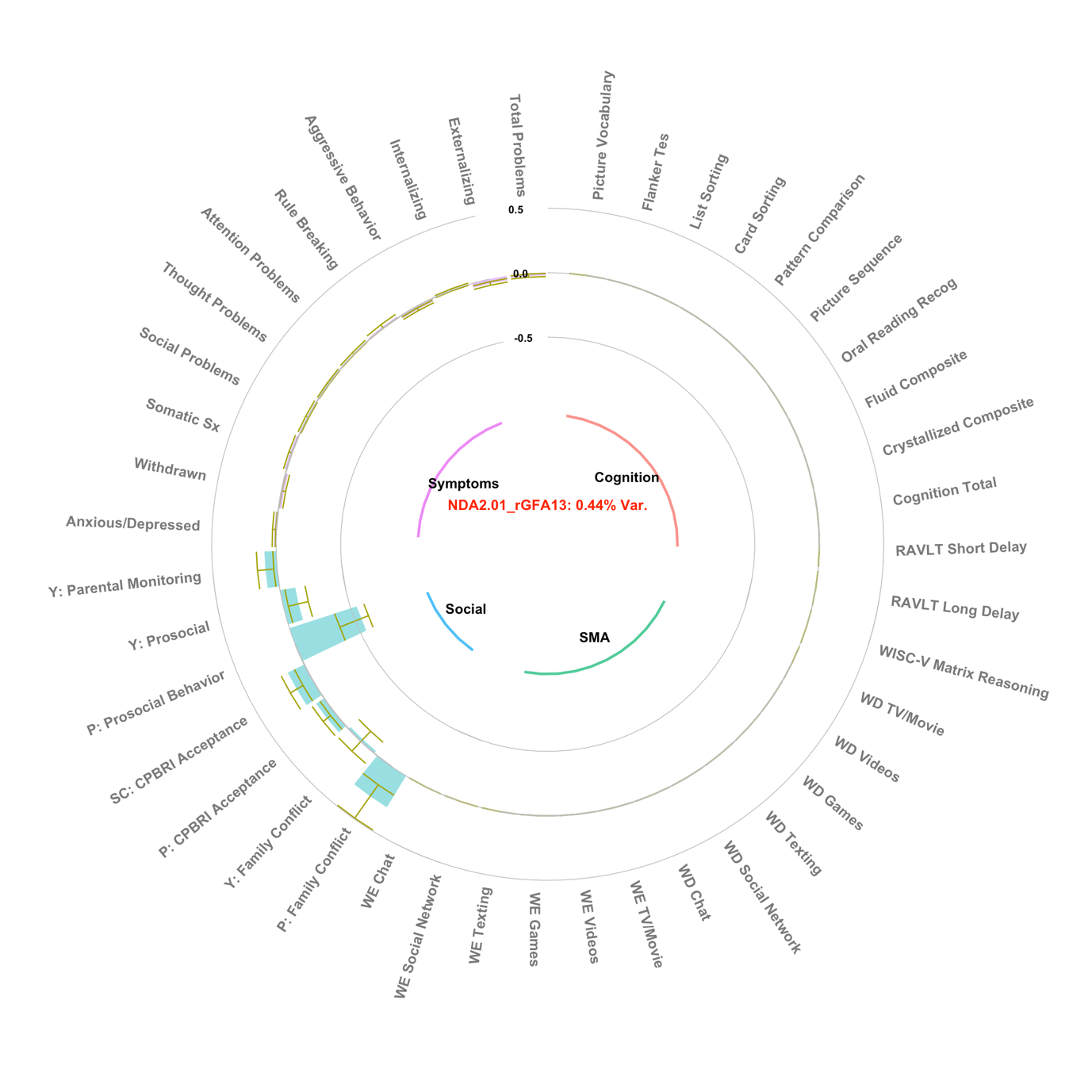
**

**Suppl Figure 15: Group factor analysis for withdrawn behavior (GF 15)**

**
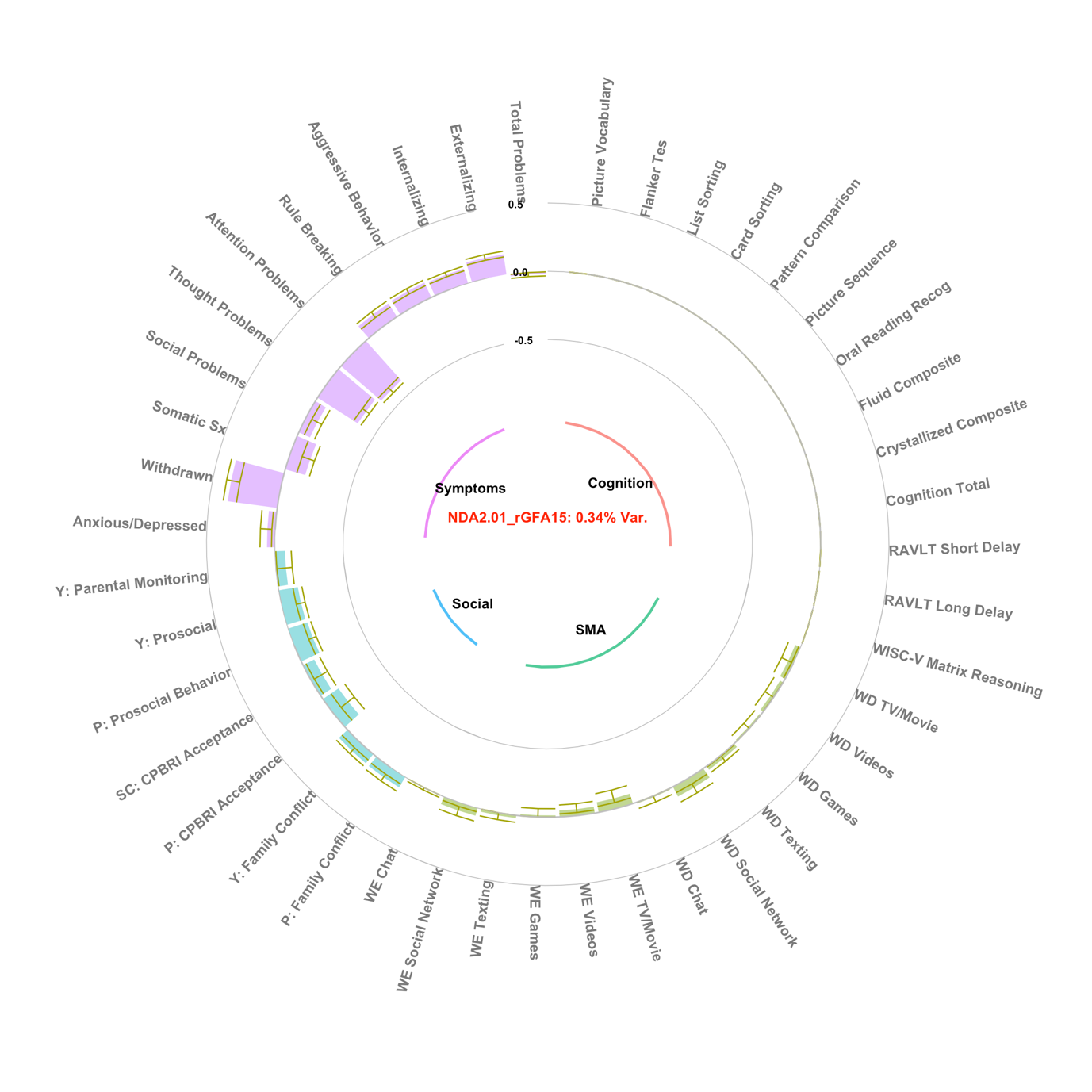
**

**Suppl Figure 16: Mixed model results for swimming/water polo**

**
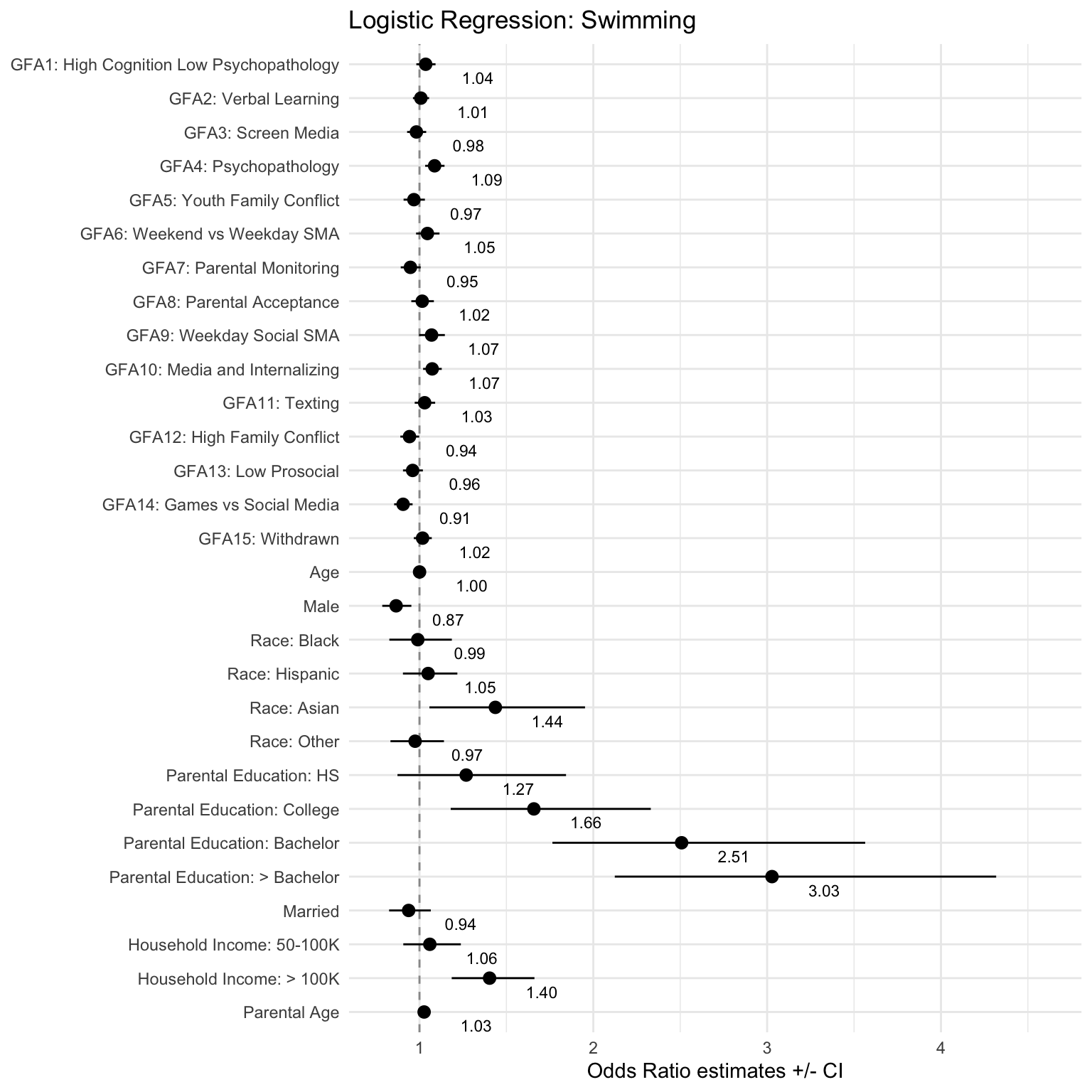
**

| *Model comparison* | BIC | Conditional R2 | dBIC |
| --- | --- | --- | --- |
| Base model | 11153.5 | .101 |  |
| GFA model | 11243.5 | .109 | 90.0 |

**Suppl Figure 17: Mixed model results for soccer**

**
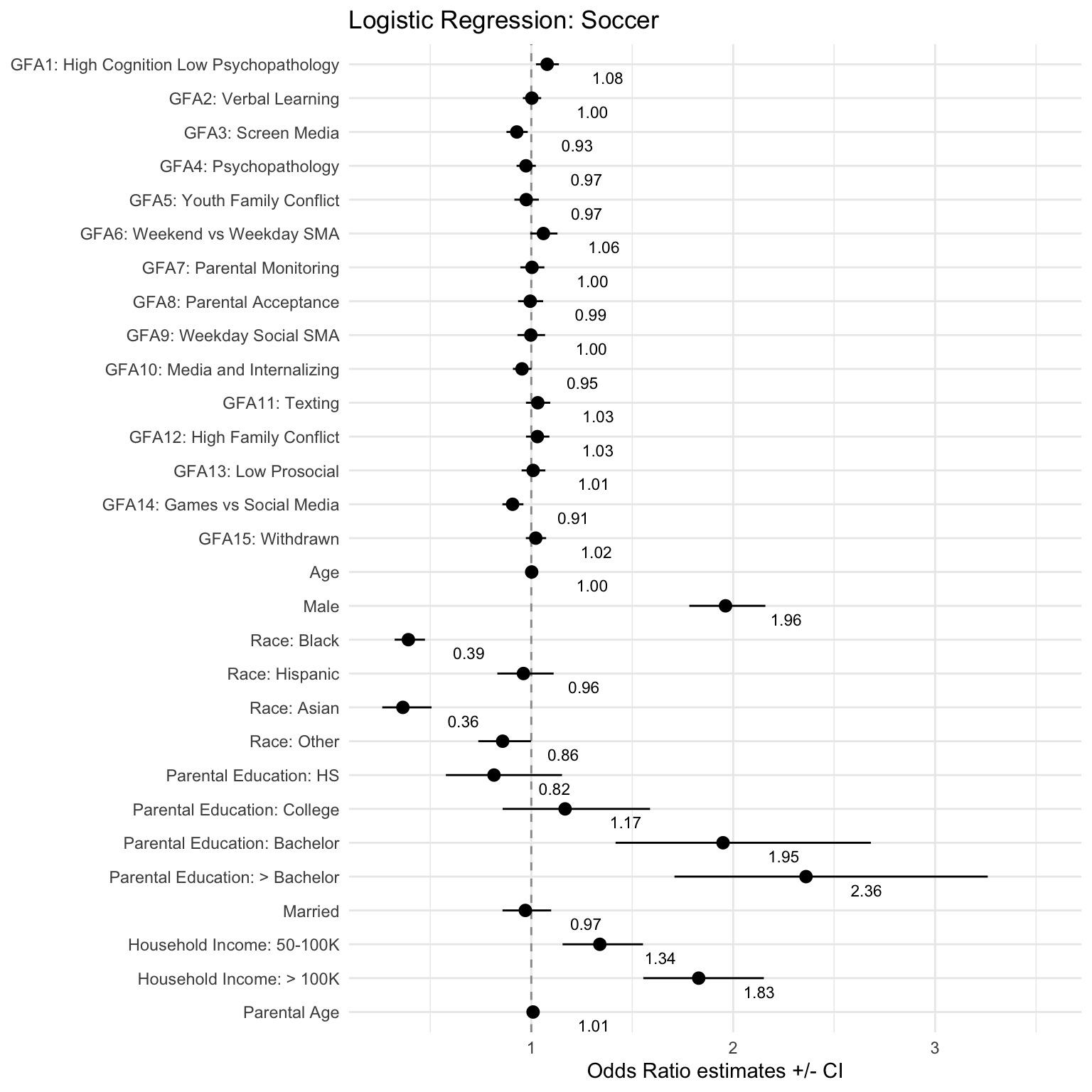
**

| *Model comparison* | BIC | Conditional R2 | dBIC |
| --- | --- | --- | --- |
| Base model | 11394.1 | .197 |  |
| GFA model | 11496.5 | .201 | 102.4 |

**Suppl Figure 18: Mixed model results for baseball**

**
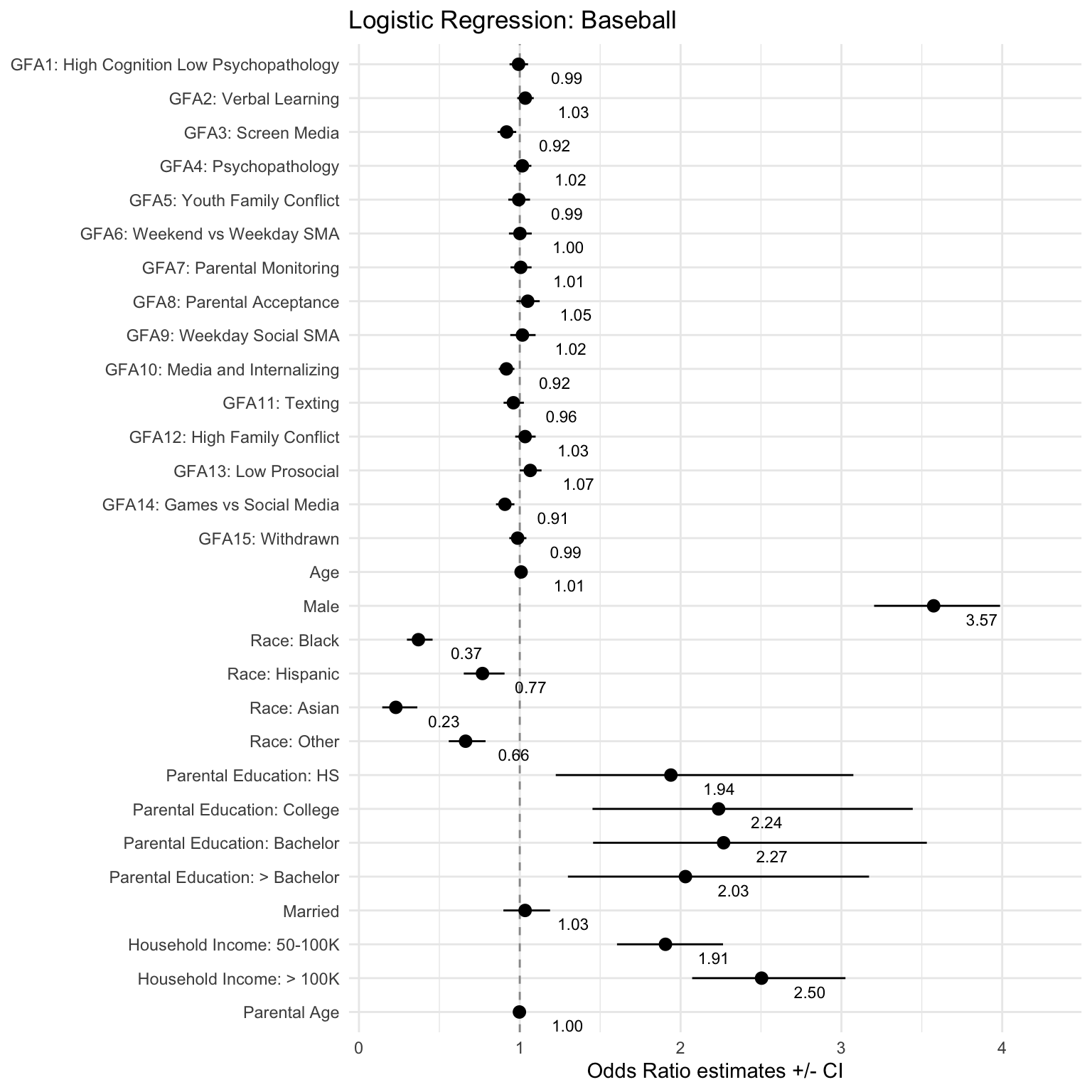
**

| *Model comparison* | BIC | Conditional R2 | dBIC |
| --- | --- | --- | --- |
| Base model | 9792.8 | .231 |  |
| GFA model | 9892.9 | .237 | 100.1 |

**Suppl Figure 19: Mixed model results for music**

**
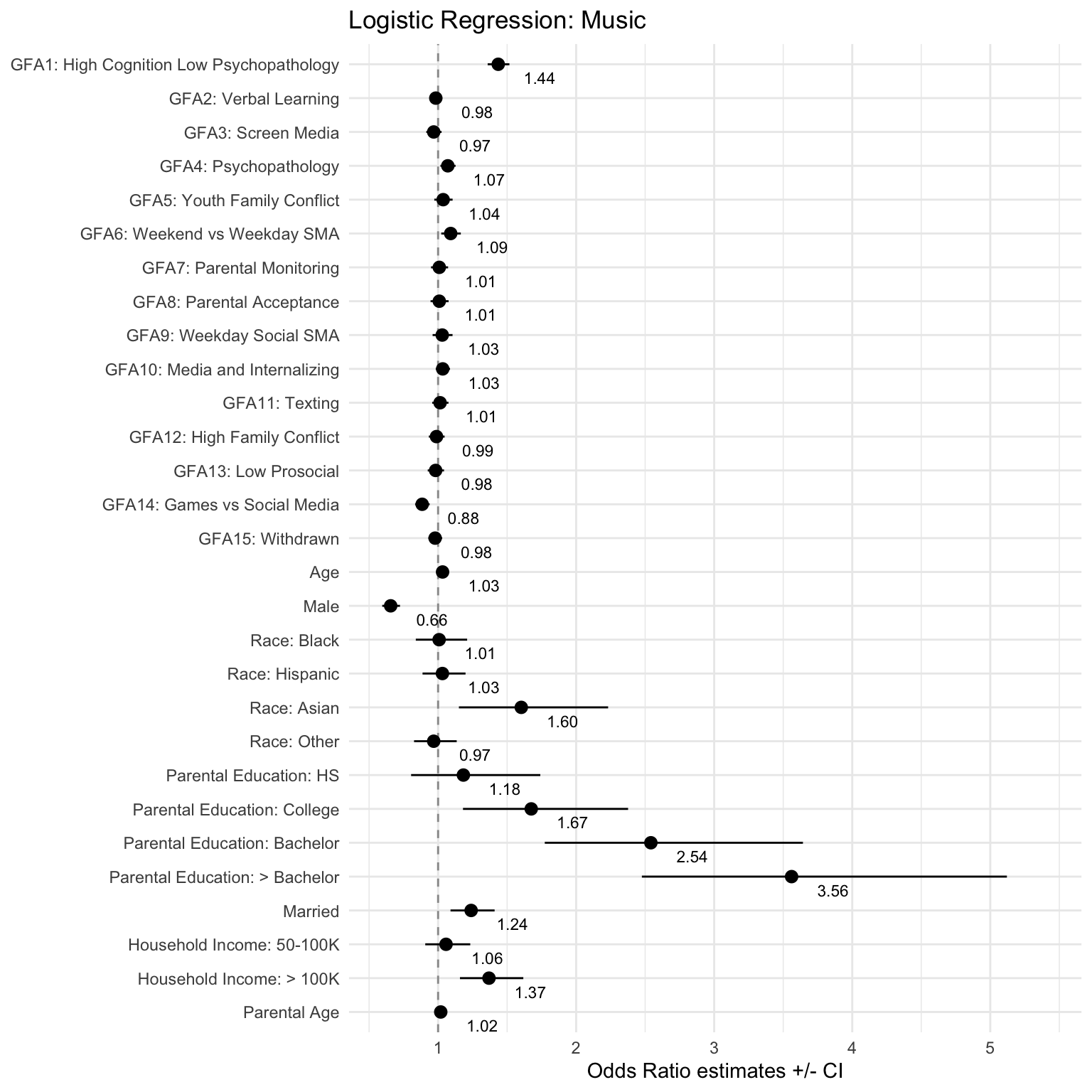
**

| *Model comparison* | BIC | Conditional R2 | dBIC |
| --- | --- | --- | --- |
| Base model | 11078.4 | .207 |  |
| GFA model | 11142.5 | .232 | 64.1 |

**Suppl Figure 20: Mixed model results for art**

**
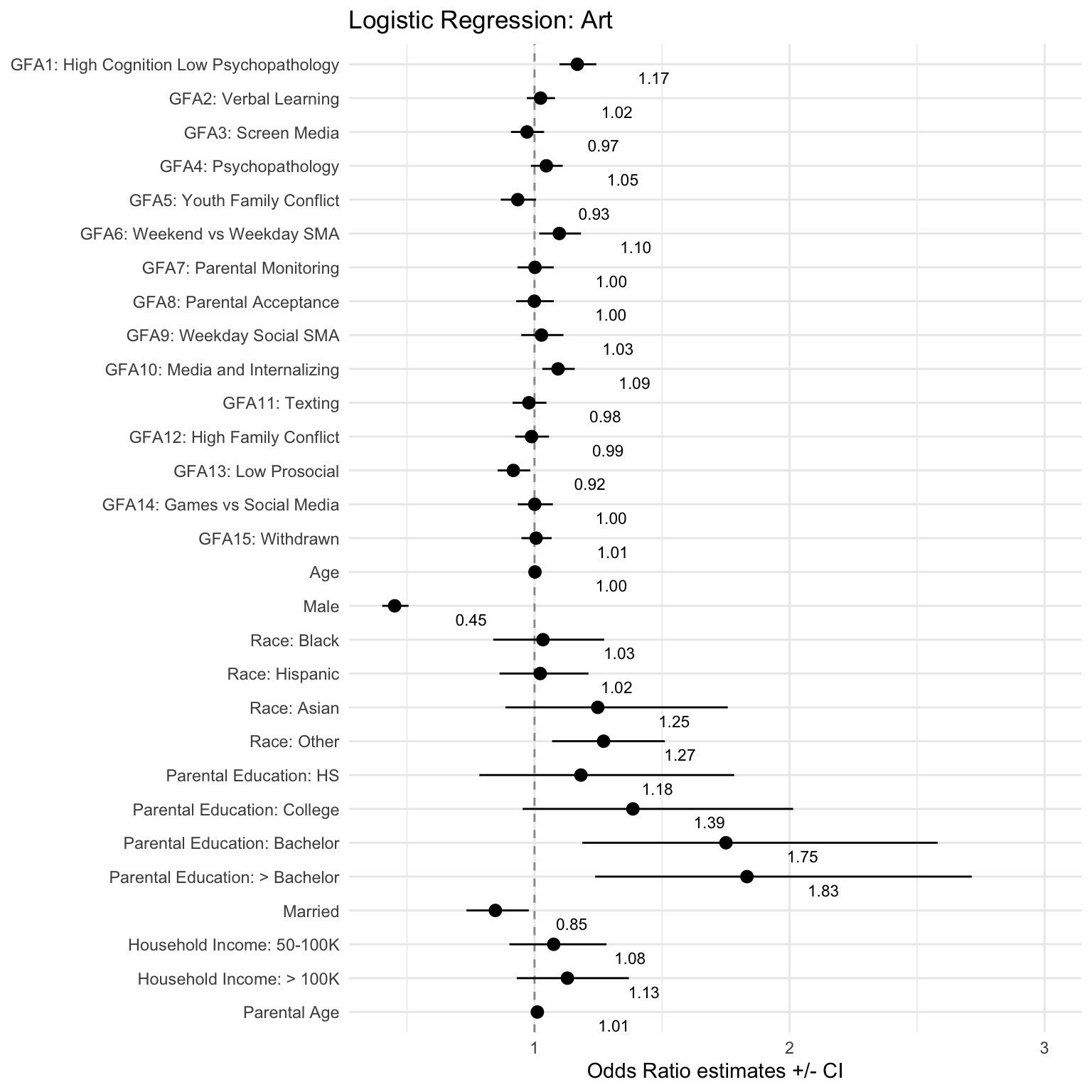
**

| *Model comparison* | BIC | Conditional R2 | dBIC |
| --- | --- | --- | --- |
| Base model | 8978.9 | .080 |  |
| GFA model | 9065.0 | .090 | 86.0 |
